# In vivo assessment of kinematic relationships for epithelial morphogenesis

**DOI:** 10.1140/epje/s10189-025-00495-2

**Published:** 2025-06-15

**Authors:** Toshinori Namba, Kaoru Sugimura, Shuji Ishihara

**Affiliations:** 1 Department of Integrated Sciences, Graduate School of Arts and Sciences, The University of Tokyo, Tokyo, 153-8902 Japan; 2 Universal Biology Institute, The University of Tokyo, Tokyo, 113-0033 Japan; 3 Department of Biological Sciences, Graduate School of Science, The University of Tokyo, Tokyo, 113-0032 Japan; 4 Department of Computational Biology and Medical Sciences, Graduate School of Frontier Sciences, The University of Tokyo, Chiba, 277-8561 Japan

## Abstract

**Abstract:**

Tissue growth and deformation result from the combined effects of various cellular events, including cell shape change, cell rearrangement, cell division, and cell death. Resolving and integrating these cellular events is essential for understanding the coordination of tissue-scale growth and deformation by individual cellular behaviors that are critical for morphogenesis, wound healing, and other collective cellular phenomena. For epithelial tissues composed of tightly connected cells, the texture tensor method provides a unified framework for quantifying tissue and cell strains by tracking individual cells in live imaging data. The corresponding kinematic relationships have been introduced in a hydrodynamic model that we previously reported. In this study, we quantitatively evaluated the kinematic equations proposed in the hydrodynamic model using experimental data from a growing *Drosophila* wing. To accomplish this, we introduced modified definitions of the texture tensor and confirmed that one of these modifications more accurately represents approximated cellular shapes without relying on *ad hoc* scaling factors. By utilizing the modified tensor, we demonstrated the compatibility of the strain rate tensors and the accuracy of both the kinematic and cell number density equations. These results cross-validate the modified texture analysis and the hydrodynamic model. Furthermore, the precision of the kinematic relationships achieved in this study provides a robust foundation for more advanced integration of modeling and experiment.

**Graphic abstract:**

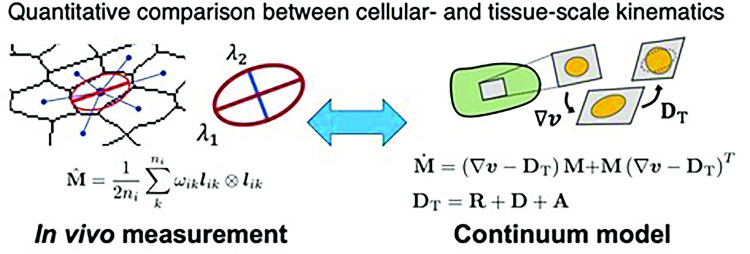

**Supplementary Information:**

The online version contains supplementary material available at 10.1140/epje/s10189-025-00495-2.

## Introduction

Morphogenesis is a self-organizing process that establishes multicellular bodies through the growth and deformation of tissues and organs. Tissue growth and deformation are driven by the behaviors of individual cells, which are coordinated through chemical and mechanical interactions among them [1–4]. Therefore, bridging the dynamics at tissue- and cell-scales is essential for understanding how precise and robust morphogenesis proceeds [4–6].

**Fig. 1:**
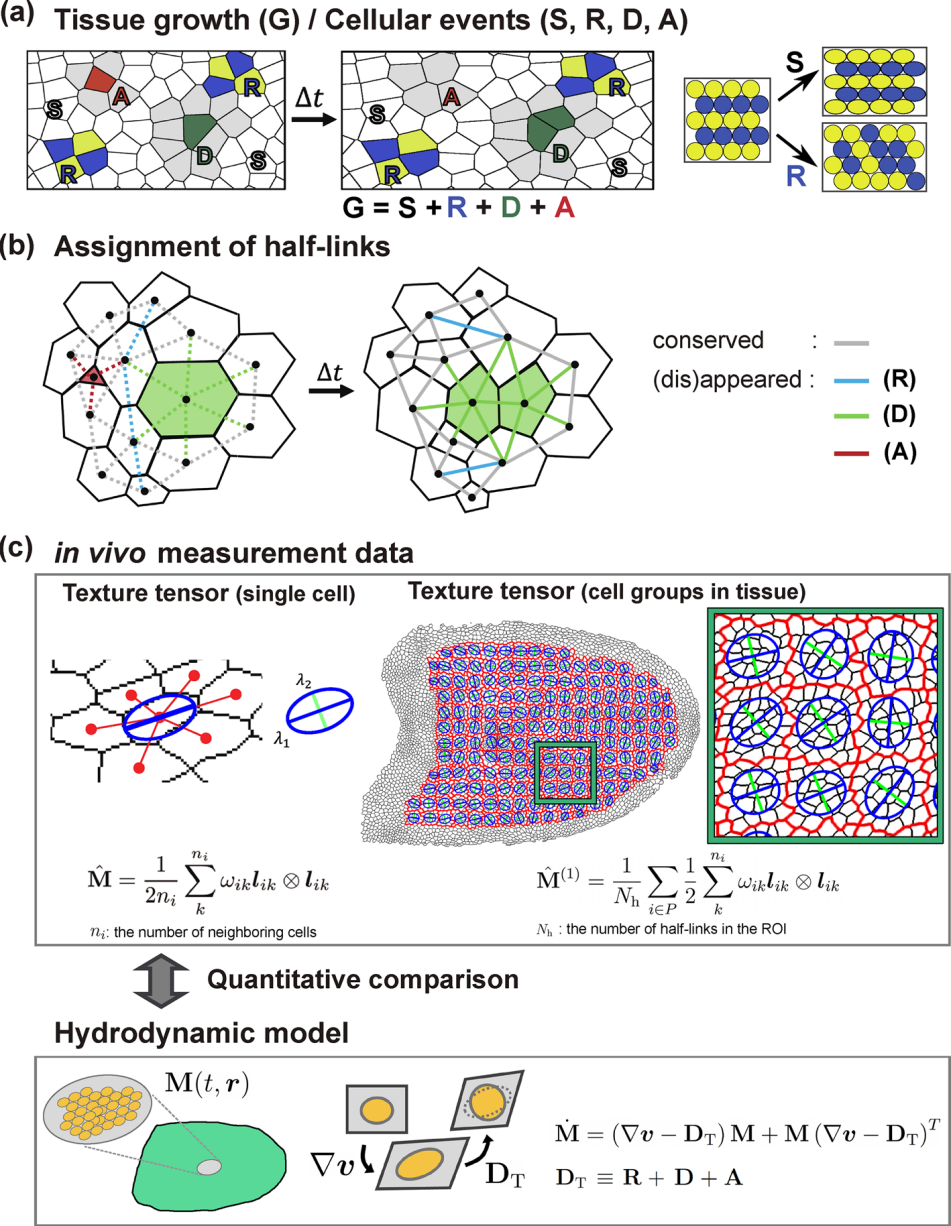
Quantification and kinematics of tissue deformation and morphogenetic cell events in epithelial tissue. **a** Morphogenetic cell events in a deforming epithelial tissue. The strain tensor $\textbf{G}$ represents horizontal elongation of tissue, where its constitutive cells undergo shape change (white; S), rearrangement (blue and yellow; R), division (green; D), and apoptosis (red; A). Division and apoptosis induce the deformation of surrounding cells (gray). Tissue deformation is the sum of strains generated by these cellular events. On the right, examples of tissue deformation driven solely by cell shape change or cell rearrangement are illustrated. **b** Assignment of cell-to-cell half-links to each morphogenetic event. The links corresponding to each event are color-coded as shown on the right. **c** Quantification (top) and kinematic equations (bottom) of tissue deformation and morphogenetic cell events. Top: The major (blue) and minor (green) axes of ellipses correspond to the eigenvectors of the texture tensor-computed for either a single cell (left; $\hat{\textbf{M}}$) or a group of cells (right; $\hat{\textbf{M}}^{(1)}$). Red outlines denote the boundaries of regions of interest (ROIs) used to compute the coarse-grained texture tensor $\hat{\textbf{M}}^{(1)}$. See also Fig. S1 for the data analysis workflow. Bottom: Kinematic equations in the multi-scale continuum model for epithelial mechanics [19] indicate that the time evolution of the cell shape tensor $\textbf{M}$ results from the tissue strain rate $\nabla \varvec{v}$ and the topological strain $\textbf{D}_\textrm{T} = \textbf{R}+\textbf{D}+\textbf{A}$

Epithelial tissues serve as excellent model systems for studying the mechanical control of morphogenesis because their relatively simple geometry and role in covering the body/organ surface make them accessible to live imaging and force/stress measurement [7–9]. Since epithelial cells are tightly connected and forces are transmitted along the plane of adherence junctions, a monolayer epithelium can be approximated as a two-dimensional tile of polygonal cells [10–12]. In such cohesive systems, tissue strain can be decomposed into strains resulting from various morphogenetic cell events, such as changes in cell shape, relative positions of cells (cell rearrangement), and cell numbers (cell division, and cell death or apoptosis) (Fig. 1a). Mathematical frameworks have been developed to quantify this decomposition of tissue strain into morphogenetic cell events (Fig. 1b, c) [13–17]. These strains were quantified as tensor variables and calculated from the experimentally observed cell geometry, providing a unified framework for quantifying strains resulting from various morphogenetic cell events, all within the same physical dimension. Along with strain measurement methods, corresponding continuum models have also been developed by coarse-graining cell-scale strains and stresses using a hydrodynamic description (Fig. 1c)  [18–28]. These measurement and modeling techniques have begun to reveal the multi-scale integration in morphogenesis, specifically how cell-scale morphogenetic events and mechanical properties contribute to tissue-scale growth and deformation [14, 15, 29–32].

A quantitative comparison of the aforementioned hydrodynamic models with *in vivo* data on cell mechanics will serve to validate the models and establish a basis for a more advanced integration of modeling and experiments. The hydrodynamic description of material deformation generally includes kinematics, kinetics, and force balance [33–35]. Kinematics describes the geometric relationships governing the time evolution of deforming objects, whereas kinetics represents the mechanical properties of materials, such as constitutive equations. Kinetics are often more intricate, involving rheology, *that is*, elasticity, viscosity/plasticity, and potentially active forces, and are often tissue-specific. In contrast, kinematic relationships provide more general and robust frameworks characterized by a few parameters, making them applicable across different epithelial tissues. Therefore, when assessing continuum models against experimental data, the kinematic relationships proposed in the theory should first be assessed.

The primary objective of this study is to quantitatively assess the kinematic equations proposed in the hydrodynamic model for epithelial mechanics [18, 19] by analyzing experimental data obtained from a developing *Drosophila* wing. To achieve this, we utilized the unified quantification method known as texture tensor analysis to measure tissue and cell strains [14]. However, the original definition of the texture tensor, used to represent the cell shape, may not be optimal for a direct comparison between the model and experimental data owing to its lack of normalization with respect to cell geometrical quantities. To address this limitation, we introduced a modified definition of the texture tensor and evaluated its effectiveness in approximating cell shape by assessing its ability to capture cell area and second moments. We also examined the compatibility of the strain rate tensor derived from texture tensor analysis with that calculated from the symmetric part of the strain tensor, as measured using particle image velocimetry (PIV), a technique used to evaluate the velocity field from images. The agreement between these two methods is a prerequisite for validating tensor analysis, but it had not been explicitly demonstrated in previous studies. By applying the modified tensor analysis to *Drosophila* pupal wing data, we demonstrated the accuracy of the kinematic equations proposed in  [18] and [19]. This, in turn, supports the validity of the tensor analysis using the modified texture tensor.

The remainder of this paper is organized as follows: Sect. 2 outlines the kinematics of tissue deformation and clarifies the purpose and problems addressed in this study. Theoretical and experimental formulations involving various tensors, such as the strain rate and cell shape tensors, are introduced. Section 3 describes the materials and methods utilized in this study. Section 4 presents the analysis results, including the introduction and evaluation of a new definition of the texture tensor, assessment of cell number density, and kinematic equations. Finally, Sect. 5 summarizes the findings and discusses their implications.

## Multi-scale hydrodynamic model and data analyses for epithelial mechanics

This section introduces kinematics in a multi-scale hydrodynamic model (Sects. 2.1 and 2.2) [19] and a related data analysis framework based on the texture tensor (Sect. 2.3) [14, 36]. Kinematic equations to be tested on experimental data are presented, along with an explanation of the methods utilized to measure kinematics from time-lapse movies of epithelial tissue.

### Strain rate tensors for tissue deformation and accompanying morphogenetic cell processes

Tissue deformation as a two-dimensional material is described by the strain rate tensor

1 $$\begin{aligned} \nabla \varvec{v} = \begin{pmatrix} \partial _x v_x & \quad \partial _y v_x \\ \partial _x v_y & \quad \partial _y v_y \end{pmatrix} \end{aligned}$$

where $\varvec{v}(t,\varvec{r})$ represents the velocity field associated with tissue deformation at time *t* and position ${\varvec{r}} = (x,y)$ (note that some literature used a transposed definition for $\nabla {\varvec{v}}$ [13, 36, 37]). The strain rate tensor is decomposed into the symmetric and asymmetric parts. The symmetric part is given by

2 $$\begin{aligned} \textbf{G} \equiv (\nabla \varvec{v} + [\nabla \varvec{v}]^{T})/2, \end{aligned}$$

where the superscript $$ ^T$$ denotes the transpose of the tensor and the asymmetric part $$\mathbf{\Omega } \equiv (\nabla \varvec{v}- [\nabla \varvec{v}]^{T})/2$$ represents rotation. Because material deformation can only be observed at discrete time intervals, data analysis should relate the strain tensor $$\nabla {\varvec{v}}$$ to the deformation that occurs during the time interval $$\Delta t$$. Let the point $${\varvec{r}}$$ at time *t* move to $${\varvec{R}}$$ at time $$t+\Delta t$$. The deformation gradient tensor $$\textbf{F}$$ between the consecutive time points is defined as follows:

3 $$\begin{aligned} \textbf{F} = {\frac{\partial {\varvec{R}}}{\partial {\varvec{r}}}}~. \end{aligned}$$

Note that ${\varvec{R}}$, and therefore $\textbf{F}$, is a function of *t* and ${\varvec{r}}$. For a sufficiently small $\Delta t$, $\textbf{F}$ is related to the strain tensor Eq. 1 as $\nabla {\varvec{v}} = (\textbf{F}-\textbf{I})/\Delta t$ with a 2 $\times$ 2 identity tensor $\textbf{I}$. The symmetric part of the strain rate tensor $\textbf{G}$ (Eq. 2) can be approximated as:

4 $$\begin{aligned} \textbf{G} \simeq \frac{1}{2\Delta t} \left( \textbf{F}^T\textbf{F}-\textbf{I} \right) ~, \end{aligned}$$

which follows from substituting the relation $\textbf{F} = \textbf{I} + \nabla {\varvec{v}} \Delta t$. This approximation becomes exact in the limit $\Delta t \rightarrow 0$.

Tissue deformation results from morphogenetic cellular events, including cell shape change (S), rearrangements (R), divisions (D), and apoptosis (A) (Fig. 1a). The tissue strain rate $\textbf{G}$ can be decomposed into the strains associated with each of these cellular events as follows:

5 $$\begin{aligned} \textbf{G} = \textbf{S} + \textbf{R} + \textbf{D} + \textbf{A} ~, \end{aligned}$$

where $\textbf{S}$, $\textbf{R}$, $\textbf{D}$, and $\textbf{A}$ represent the symmetric strain rate tensors resulting from cell shape change, rearrangement, division, and apoptosis, respectively. In the multi-scale continuum theory, the strain $\textbf{S}$ is associated with the resilient force responsible for maintaining cellular shape, while $\textbf{R}$, $\textbf{D}$, and $\textbf{A}$ represent strains that accompany changes in relative cellular positions and do not contribute to resilient forces. Thus, we refer to $\textbf{G}$ and $\textbf{S}$ as total and elastic strains, respectively, while classifying the others as plastic strains.

### Kinematic equations for two-dimensional cell tiles

The kinematic equations for a two-dimensional tile of epithelial cells were proposed as a coarse-grained representation of the deformation resulting from morphogenetic cell events [18]. Under the assumption that the cellular shape can be approximated by an ellipse, it is quantified using a 2 $\times$ 2 matrix $\textbf{M}$ [36]. The matrix $\textbf{M}$ is symmetric and positive-definite, and has dimensions of squared length. The two eigenvectors of $\textbf{M}$ point to the longer and shorter axes of the ellipse, with the corresponding eigenvalues representing the squared lengths of the radii. The cell area is approximated from the tensor $\textbf{M}$ as follows:

6 $$\begin{aligned} A = \pi \left| \textbf{M}\right| ^{1/2} \end{aligned}$$

where $\left| \textbf{M}\right|$ denotes the determinant of $\textbf{M}$. For a continuum description of multicellular tissues, the tensor field variable $\textbf{M}(t,\varvec{r})$ is introduced as a measure of the coarse-grained cell shape at time *t* and position $\varvec{r}$ (bottom panel in Fig. 1c). Because the epithelial tissue comprises a tile of cells, the evolution of cell shape over time is closely linked to tissue deformation, quantified by the velocity field $\varvec{v}(t,\varvec{r})$. Theoretical analysis led to the relationship [18, 19]

7 $$\begin{aligned} \dot{\textbf{M}} = \left( \nabla \varvec{v} - \textbf{D}_\textrm{T} \right) \textbf{M} + \textbf{M} \left( \nabla \varvec{v}-\textbf{D}_\textrm{T}\right) ^T~, \end{aligned}$$

where $\dot{\textbf{M}} \equiv \partial _t \textbf{M} + \varvec{v} \cdot \nabla \textbf{M}$ represents the Lagrange derivative of $\textbf{M}$ and $\textbf{D}_\textrm{T} \equiv \textbf{R} +\textbf{D} +\textbf{A} =\textbf{G} -\textbf{S}$ denotes a strain rate tensor resulting from morphogenetic cell events accompanied by topological changes in cell-neighbor relationships. This equation is hereafter referred to as the kinematic equation for $\textbf{M}$. The equation comprises no mechanical parameters and must be satisfied if the tensors $\textbf{M}$ and $\textbf{D}_\textrm{T}$ are appropriately defined and measured from the image data.

Cell number density changes owing to alterations in cell shape, division rates, influx, and efflux. Cell number density is simply the inverse of cell area, expressed by $\rho = 1/A = 1/\pi \!\left| \textbf{M}\right| ^{1/2}$. Using the identity $d|\textbf{a}|/dt = |\textbf{a}| \textrm{Tr}\left( \textbf{a}^{-1} d\textbf{a}/dt\right)$ for an arbitrary regular matrix $\textbf{a}$ that is a function of a variable *t*, the equation for changes in cell number density is derived from Eq. 7 as follows:

8 $$\begin{aligned} \frac{\partial \rho }{\partial t} + \nabla \cdot \left( \rho \varvec{v} \right) = (\textrm{Tr}  \textbf{D}_\textrm{T}) \rho \end{aligned}$$

where $\textrm{Tr}  \textbf{D}_\textrm{T}$ denotes the trace of $\textbf{D}_\textrm{T}$. This equation represents the cell number conservation over time, where $\textrm{Tr}  \textbf{D}_\textrm{T}$ corresponds to the variation rate of cell number density. Similar to Eq. 7, the equation contains no mechanical parameters and should be satisfied accordingly.

The trace of the strain rate tensor $\nabla {\varvec{v}}$ represents the local rate of area change (i.e., expansion or contraction) at that point. Because cell rearrangement does not contribute to area changes, the following assumption should apply:

9 $$\begin{aligned} \textrm{Tr} \textbf{R} = 0. \end{aligned}$$

This indicates that $\textbf{R}$ is not involved in the variation rate of cell number density. Traces of cell division and apoptosis tensors, $\textbf{D}$ and $\textbf{A}$, correspond to cell division and death rates, respectively, with anticipated values $\textrm{Tr}  \textbf{D} \ge 0$ and $\textrm{Tr}  \textbf{A} \le 0$.

Notably, the derivation of the cell number density equation in Eq. 8 is a natural outcome of the kinematic equation for $\textbf{M}$ (Eq. 7) [18, 19]. This integration aligns with the characteristics of tissue, where changes in cell shape are inherently linked to tissue deformation (see also the discussion in Sect. 5).

### Coarse-grained and unified quantification of morphogenetic cell events using the texture tensor

Experimental measurement of the quantities explained above is not a trivial problem, for which one needs to properly define the cellular shape and its temporal variation, and their decomposition in accordance with Eq. 5. The texture tensor, a 2$\times$2 matrix representing coarse-grained cell shape, can be obtained from experimental data including links between the centers of neighboring cells (Fig. 1b, c). The texture tensor was originally defined in [14, 36] as follows:

10 $$\begin{aligned} \hat{\textbf{M}}^{(0)} = \sum _{i \in P}\frac{1}{2} \sum _{k}^{n_i} \omega _{ik}\varvec{l}_{ik} \otimes \varvec{l}_{ik}~, \end{aligned}$$

where, $\varvec{l}_{ik} = \varvec{x}_k-\varvec{x}_i$ denotes the vector connecting the cell centers of cell *i* and its neighboring cell *k*. The hat symbol $\hat{~\cdot ~}$ indicates a tensor derived from half-links $\varvec{l}_{ik}$ (this convention applies throughout this manuscript). The sum of $\omega _{ik} {\varvec{l}}_{ik} \otimes \varvec{l}_{ik}$ is computed over cells in the region of interest (ROI) indexed by *P*. The sum over *k* indicates the sum over the links connecting cell *i* with its neighboring cells, where $n_i$ represents the number of neighboring cells. The weighting parameter $\omega _{ik}$ is set to $\omega _{ik}=1$ if neighboring cells are in contact with the edge, and $\omega _{ik} = 1/2$ if they are in contact with the fourfold vertex. Because each link was counted twice, a factor 1/2 is applied, each link $\varvec{l}_{ik}$ is thus designated as a half-link. The half-links to and from the outmost cells are excluded when calculating $\hat{\textbf{M}}^{(0)}$.

By using the temporal change in the texture tensor $\hat{\textbf{M}}^{(0)}$, Guirao et al. [14] developed a mathematical framework for calculating the total strain $\textbf{G}$ and the decomposition to $\textbf{S}$, $\textbf{R}$, $\textbf{D}$, and $\textbf{A}$ to align with Eq. 5 (Fig. 1c and Fig. S1; Supporting Information A). In this framework, the deformation gradient tensor of ROI, denoted as $\hat{\textbf{F}}$, is computed from the difference in $\hat{\textbf{M}}$ between consecutive time points *t* and $t+\Delta t$ (Fig. S1d). While this framework can accommodate data in both Eulerian and Lagrangian descriptions, our previous and current studies have employed Lagrangian tracking (i.e., the individual cells are tracked from the initial to the final time points) [14]. This approach allows us to define ROIs with one-to-one correspondence across time frames (Sect. 3.7), enabling the evaluation of temporal changes in physical quantities between time points *t* and $t+\Delta t$, while excluding the effects of influx and efflux, and facilitating the computation of their Lagrange derivatives (see Sect. 3.8).

Subsequently, the symmetric part of the strain tensor, $\hat{\textbf{G}}$, is determined using Eq. 4, as follows:

11 $$\begin{aligned} \hat{\textbf{G}} = \frac{1}{2\Delta t} \left( \hat{\textbf{F}}^T \hat{\textbf{F}}- \textbf{I}\right) ~. \end{aligned}$$

Simultaneously, by tracking the appearance and disappearance of half-links, the decomposition of $\hat{\textbf{G}}$ into $\hat{\textbf{S}}$, $\hat{\textbf{R}}$, $\hat{\textbf{D}}$, and $\hat{\textbf{A}}$ is computed from cellular contour images to satisfy

12 $$\begin{aligned} \hat{\textbf{G}} = \hat{\textbf{S}} + \hat{\textbf{R}} + \hat{\textbf{D}} + \hat{\textbf{A}}~, \end{aligned}$$

corresponding to Eq. 5.

The details of this method are summarized in Supporting Information A and B. Our method has been slightly modified from the original to more directly calculate $\hat{\textbf{G}}$, with the difference remaining small-on the order of $\mathcal {O}(\Delta t^2)$ (Supporting Information A). Additionally, extracting strain rate tensors from experimental data involves different assignment options, even when skeletonized images are provided. The original assignment rules [14] were validated as outlined in detail (Supporting Information B).

While the method requires cell contour data from time-lapse images to compute $\hat{\textbf{G}}$ and its decomposition $\hat{\textbf{S}}, \hat{\textbf{R}}, \hat{\textbf{D}}$, and $\hat{\textbf{A}}$, the strain rate tensor $\textbf{G}$ can also be determined from the velocity field of a tissue, which can be obtained using less intensive image processing methods such as PIV. Comparison of strain rate tensors obtained by the two different measurement is performed in Sect. 4.3.

## Materials and methods

### *Drosophila* genetics

*Drosophila* stocks were maintained at $25^\circ$C. The genotype of the three wild-type samples utilized in this study is DE-cad-GFP [38].

### Image acquisition

The preparation of *Drosophila* pupal wing samples for image collection was conducted following previously established protocols [14, 39]. Briefly, pupae at appropriate developmental stages were dissected to remove the pupal case covering the left wing and were then positioned on a small drop of Immersol W 2010 (Zeiss 444969-0000-000) in a glass bottom dish. Images were acquired at $25^\circ$C using an inverted confocal spinning disk microscope (Olympus IX83 combined with Yokogawa CSU-W1) equipped with an iXon3 888 EMCCD camera (Andor), an Olympus 60x/NA1.2 SPlanApo water-immersion objective, and a temperature control chamber (TOKAI HIT), utilizing IQ 2.9.1 (Andor) [14].

### Image data

**Fig. 2:**
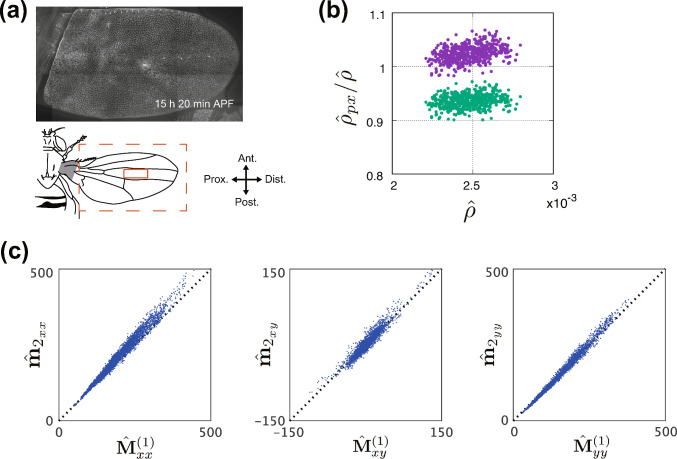
Validation of the texture tensor $\hat{\textbf{M}}^{(1)}$ as a cell shape indicator. **a** Top: Image of DE-cad-GFP from the *Drosophila* pupal wing at 15 h 20 min APF. Bottom: Schematic of the *Drosophila* pupal wing. We analyzed images of the whole-wing (black orange square) and the C region (orange square). **b** Ratio of cell number density measured by direct pixel counting ($\hat{\rho }_{px}$) to that measured by the texture tensor ($\hat{\rho }$) is shown. Direct pixel counting of $\hat{\rho }_{px}$ was performed both excluding the outline pixels (purple points) and including half of the outline pixels (green points). Each data point represents the ratio value for a single ROI in the C region. **c**Comparison between the texture tensor $\hat{\textbf{M}}^{(1)}$ (Eq. 13) and the mean second moment of cells, $\hat{\textbf{m}}_{2}$ (Eq. 15). Each component of these tensors is indicated in the respective panels

Two types of image data were analyzed (Fig. 2a): 1. Whole-wing images acquired at 5-minute intervals starting from 15.5 h after puparium formation (h APF). 2. C region images acquired at 1-minute intervals starting from 24 h APF. One of the three former type images was published in [14], and all three of the latter type images were examined in [40].

### Image processing

Image segmentation and tiling were performed using custom-made macros and plug-ins in ImageJ [14, 41]. Vertex position and connectivity were extracted from the skeletonized images using custom-made code in OpenCV [41]. See also Fig. S1 for the data analysis workflow.

### Particle image velocimetry (PIV)

The displacement within sequential images was measured using **PIVlab**, an open-source tool in MATLAB [42], with a correlation algorithm *FFT window deformation* (direct Fourier transform correlation with multiple passes and deforming windows), along with ensemble correlation. The interrogation window and window steps were set at 64$\times$64 and 16 pixels, respectively. Erroneous vectors were eliminated and supplemented by the standard deviation filter with a parameter of $n=3$.

### Cell tracking

The cell IDs were assigned by labeling each segmented area of the skeletonized images using the function **bwlabeln** in MATLAB. From the assigned cell IDs, the IDs of all neighboring cells were obtained, with cells adjacent to the outer region designated as the outermost cells. Subsequently, a cell tracking procedure was implemented. Initially, the *x*-*y* coordinates of the cell center were adjusted based on the vector field measured using PIV. Second, a cost matrix, defined by the square Euclidean distance between cell centers, was optimized using the function **assignjv** in MATLAB to determine the correspondence of cell IDs in two consecutive time frames [43]. To reduce the computational cost, the components of the cost matrix for cell pairs over 400 pixels apart were set to infinity. Third, cell division and apoptosis were detected based on the appearance and disappearance of cells, respectively. We examined the changes in the area of neighboring cells surrounding newly emerged cells, designating the cell with the largest decrease in area as a dividing cell. While this method may not be effective in cases where neighboring cells undergo division and apoptosis simultaneously, such occurrences were rare in the time-lapse images analyzed in this study. We thoroughly reviewed all cell tracking patterns and made manual adjustments as needed.

### Setting ROI

To calculate texture tensors and strain rates from the time-lapse movies of the pupal wing, the image at the initial time point, either 15.5 h APF (whole-wing movie) or at 24 h APF (C region movie), was divided into 120$\times$120 pixel tiles. ROIs were then defined as regions composed of cells approximating these rectangular tiles (Fig. S1d). Each ROI defined at the initial frame was tracked over time to determine the corresponding ROIs in subsequent frames. Thus, ROIs across different time points maintain mutual correspondence—that is, they are composed of the same cells, or of their mother or daughter cells. An ROI of this size contains approximately 30 cells at 15.5 h APF and approximately 60 cells at 32 h APF. Previous research has shown that this ROI size produces consistent patterns of tissue deformation, cellular events, and stress in the *Drosophila* pupal wing and notum [14, 44].

### Computation of time derivative of coarse-grained quantities

Coarse-grained quantities are defined using the ROIs described in Sect. 3.7. Temporal changes in physical quantities are calculated from corresponding ROIs between consecutive time points *t* and $t+\Delta t$ (Fig. S1c), allowing for the evaluation of Lagrange derivatives; for example, $\Delta \hat{\textbf{M}}/\Delta t$ serves as a measure of the Lagrange derivative $\dot{\textbf{M}} \equiv \partial _t \hat{\textbf{M}} + {\varvec{v}}\cdot \nabla \hat{\textbf{M}}$. For the calculation of time derivatives and the estimation of $\hat{\textbf{F}}$, we assumed that the deformation within each ROI is sufficiently small and that most half-links are conserved between time points *t* and $t + \Delta t$. In our analysis, a 5-minute time interval satisfies this assumption. As confirmed in the Results (Sect. 4), second-order terms in $\Delta t$ are negligible, supporting the validity of this approximation

### Assignment of half-links to cellular events

We implemented the methodology outlined in Ref. [14] to assign each half-link to one of the cellular events, such as cell shape change, rearrangement, division, and apoptosis (Fig. 1b; refer to Supporting Information B for details).

## Results

### Modified definitions of the texture tensor

The original texture tensor, defined in Eq. 10, $\hat{\textbf{M}}^{(0)}$, exhibits a scaling behavior with the number of cells within the ROI, $N_\textrm{c}$. This dependence on the choice of ROI is incompatible with the definition of cell shape tensor used in the hydrodynamic model (Sect. 2.2) [19], which is based on the coarse-grained representation of cell shapes and remains invariant to the number of cells within the ROI. We therefore introduced an alternative form of the texture tensor as follows:

13 $$\begin{aligned} \hat{\textbf{M}}^{(1)}&= \frac{1}{N_\textrm{h}} \sum _{i \in P} \frac{1}{2} \sum _{k}^{n_i} \omega _{ik}\varvec{l}_{ik} \otimes \varvec{l}_{ik}~, \end{aligned}$$

where $N_\textrm{h}$ denotes the number of half-links in the ROI. In this definition, the texture tensor is normalized by $N_\textrm{h}$ rather than being proportional to $N_\textrm{c}$. Furthermore, we also considered other possible forms of the texture tensor ($\hat{\textbf{M}}^{(2)}$, $\hat{\textbf{M}}^{(3)}$, and $\hat{\textbf{M}}^{(4)}$, in Supporting Information C). All the proposed definitions of texture tensors possess a physical dimension of squared length, differing primarily in the normalization procedure based on the number of cells and their adjacent counterparts.

### Evaluation of the cellular area and second moment using the modified texture tensors

To assess the accuracy of the modified texture tensors in capturing the cell morphology within epithelial tissues, we conducted a comparative analysis of the cellular area and second moment measurements obtained using different texture tensor formulations against those derived directly from pixel counting in image data of the *Drosophila* pupal wing (Fig. 2). The ratio of cell number density measured by direct pixel counting to that measured by the texture tensor $\hat{\textbf{M}}^{(1)}$ is shown in Fig. 2b, where the individual dots represent data from single ROIs of 120 pixels $\times$ 120 pixels, with averages computed across ROIs of this specific size, unless otherwise specified hereinafter. The ratio exceeded unity when excluding pixels along cell contours (Fig. 2b, magenta dots) but decreased when half of the pixels on the cell contours were included (Fig. 2b, green dots). These results suggest that the modified texture tensor $\hat{\textbf{M}}^{(1)}$ accurately measures the cell area, with deviations from unity attributed to image resolution in the measurement.

Next, we compared $\hat{\textbf{M}}^{(1)}$ with the second moment of the cellular shape, quantified using pixel counting. The second moment of a cell, based on pixel data defining cellular shape, is calculated as follows:

14 $$\begin{aligned} \hat{\textbf{m}}_{2c} = \frac{1}{A_\textrm{px}} \begin{pmatrix} \sum \left( x_i-x_c\right) ^2 &  \sum \left( x_i-x_c\right) \left( y_i-y_c\right) \\ \sum \left( x_i-x_c\right) \left( y_i-y_c\right) &  \sum \left( y_i-y_c\right) ^2 &  \end{pmatrix} \end{aligned}$$

where the sum is taken over all pixels within the cell, with each pixel located at $(x_i, y_i)$. The coordinates $(x_c, y_c)$ represent the center of the cell (e.g., $x_c = \frac{1}{A_\textrm{px}}\sum x_i$) and $A_\textrm{px}$ denotes the cell area as determined by pixel counting. We averaged the second moment of the cells over the ROI and calculated

15 $$\begin{aligned} \hat{\textbf{m}}_2 = \frac{4}{N_\textrm{c}}\sum _{c \in \mathrm ROI} \hat{\textbf{m}}_{2c}~. \end{aligned}$$

We applied a factor of 4 because an ellipse with radii $r_a$ and $r_b$ along the *x*- and *y*-axes has second moments $m_{xx} = r_a^2/4$, $m_{yy} = r_b^2/4$, and $m_{xy} = 0$. Therefore, $\pi |\hat{\textbf{m}}_2|^{1/2}$ corresponds to the area, which is consistent with Eq. 6. As shown in Fig. 2c, the texture tensor $\hat{\textbf{M}}^{(1)}$ closely aligns with the pixel-based mean second moment of the cellular shape in each ROI, $\hat{\textbf{m}}_2$. These findings collectively demonstrate that $\hat{\textbf{M}}^{(1)}$ accurately measures cellular area and second moment from real image data.

For comparison, we assessed alternative definitions of the texture tensors against the second moments of the cellular shape, $\hat{\textbf{m}}_2$. We plotted $\hat{\textbf{M}}^{(m)}$ ($m = 0,2,3,4$) and $\alpha \hat{\textbf{m}}_2$ in Figs. S2a–d, where the scaling factor $\alpha$ is introduced as a fitting parameter determined by the mean value of the cell area. $\hat{\textbf{M}}^{(0)}$ and $\hat{\textbf{M}}^{(3)}$ did not align with the second moment $\hat{\textbf{m}}_2$ (Figs. S2a and c). This discrepancy stemmed from the fact that they were not normalized by the cell number $N_\textrm{c}$, which varies across the dataset. This is in sharp contrast to $\hat{\textbf{M}}^{(1)}$, which only requires the predetermined factor of 4. $\hat{\textbf{M}}^{(2)}$ and $\hat{\textbf{M}}^{(4)}$ aligned significantly, comparable with $\hat{\textbf{M}}^{(1)}$. The scaling factor was $\alpha \sim 5.8$ for $\hat{\textbf{M}}^{(2)}$, which can be attributed to the fact that $\hat{\textbf{M}}^{(2)}$ differed from $\hat{\textbf{M}}^{(1)}$ only in the scaling ratio $N_\textrm{h}/N_\textrm{c}$, which is expected to be close to 6 (Fig. S2b). $\hat{\textbf{M}}^{(4)}$ showed results almost identical to $\hat{\textbf{M}}^{(1)}$ with a similar scaling factor $\alpha = 0.95$ (Fig. S2d).

### Consistency in the strain rate measured using $\hat{\textbf{G}}$ and PIV

In Sect. 4.2, we validated the utilization of the modified texture tensor $\hat{\textbf{M}}^{(1)}$ to measure the morphology of cells using still images. Below, we assessed its ability to measure the dynamic deformation of tissues. The deformation gradient tensor $\hat{\textbf{F}}$ and the symmetric part of the strain rate tensor $\hat{\textbf{G}}$ were calculated from the time variation of the texture tensor, which requires segmented images that highlight cell contours. However, the strain rate tensor $\nabla {\varvec{v}}$ can also be determined by PIV without image segmentation, providing a direct characterization of tissue deformation. These two quantities $\hat{\textbf{F}}$ and $\nabla {\varvec{v}}$ should be related to $\nabla \varvec{v} = (\hat{\textbf{F}}-\textbf{I})/\Delta t$, assuming a sufficiently small time interval $\Delta t$. Ensuring consistency between the left- and right-hand sides obtained using different measurement methods is a prerequisite for validating the analysis based on the texture tensor.

**Fig. 3:**
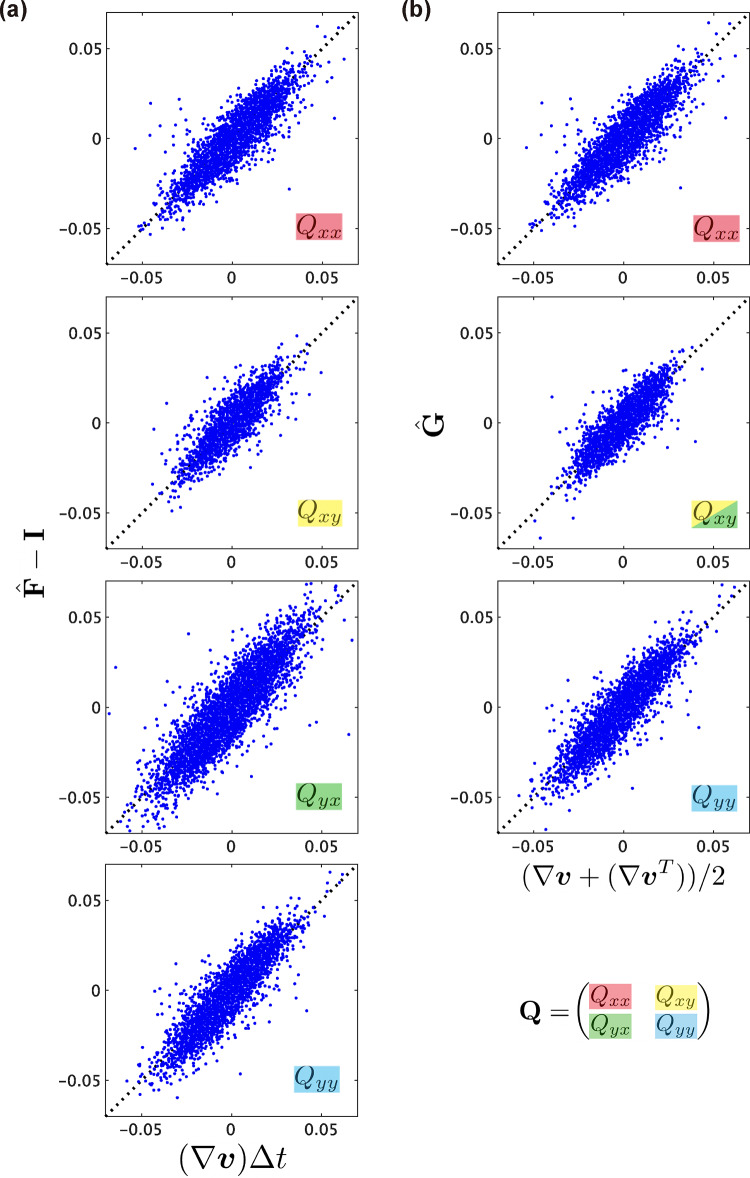
Validation of the strain rate measurement. **a** Comparison of the strain rate tensor $\nabla {\varvec{v}}$ and deformation gradient tensor $\hat{\textbf{F}}$. The average of the vectors obtained by PIV over the ROI was used to compute the strain rate tensor $\nabla {\varvec{v}}$. **b** Comparison of the components of $\hat{\textbf{G}}$ with the symmetric part of $\nabla {\varvec{v}}$ obtained from PIV

As shown in Fig. 3a, $(\nabla \varvec{v})\Delta t$ measured using PIV and $\hat{\textbf{F}}-\textbf{I}$ calculated from the texture tensor $\hat{\textbf{M}}^{(1)}$ agreed with each other (see Fig. S3 for results from different samples of whole-wing image data). Moreover, when $\Delta t$ is small, $\hat{\textbf{G}}$ can be approximated by the symmetric part of $\nabla {\varvec{v}}$ (Eq. 2), a relationship validated in Fig. 3b and Figs. S4. In our dataset, $\Delta t = 5$ min, the relative magnitude of the discrepancy between Eq. 11 and Eq. 2 is $\sim 10^{-2}$ times smaller than that of the symmetric term, which makes it negligible. These findings validate $\hat{\textbf{F}}$ and $\hat{\textbf{G}}$ derived from $\hat{\textbf{M}}^{(1)}$ as indicators of dynamic tissue deformation.

### Assessment of the cell number density equation

Having confirmed the applicability of the modified texture tensor $\hat{\textbf{M}}^{(1)}$ for both static cell shape analysis (Sect. 4.2) and dynamic deformation analysis (Sect. 4.3), we next investigated whether the time evolution equation for cell number density (Eq. 8) in our continuum model aligned with the *in vivo* data obtained using $\hat{\textbf{M}}^{(1)}$.

**Fig. 4:**
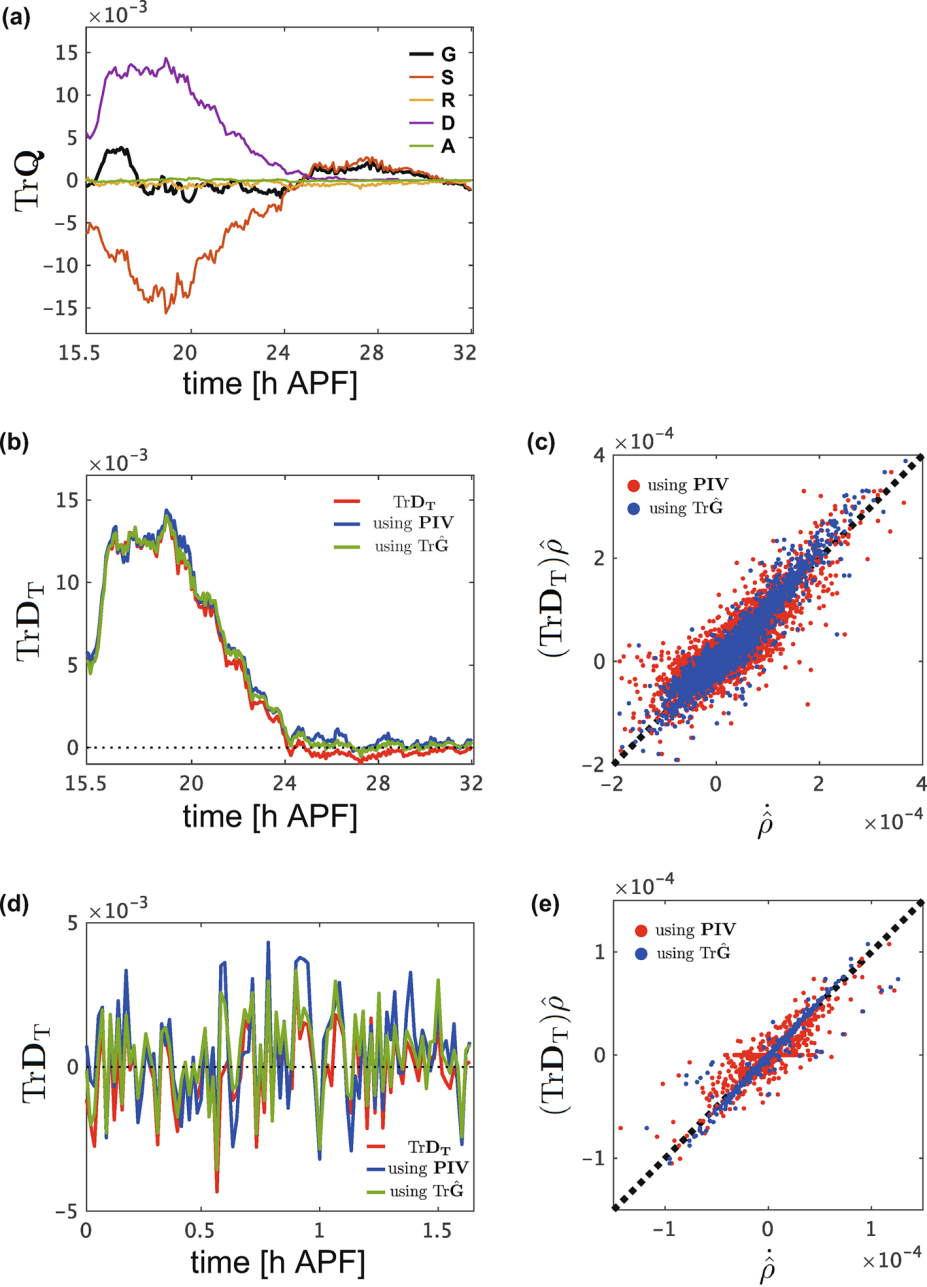
Assessment of the cell number density equation. **a** Time series of the traces of $\textrm{Tr}{\hat{\textbf{G}}}$ (black), $\textrm{Tr}{\hat{\textbf{S}}}$ (red), $\textrm{Tr}{\hat{\textbf{R}}}$ (yellow), $\textrm{Tr}{\hat{\textbf{D}}}$ (purple), and $\textrm{Tr}{\hat{\textbf{A}}}$ (green) for time-lapse data of the whole-wing captured at 5-min intervals starting from 15 h 30 min APF. **b**, **d** Time series data indicating the value obtained by dividing the value on the left-hand side of Eq. 8 by $\hat{\rho }$ (blue and green lines), compared with $\textrm{Tr}  \textbf{D}_\textrm{T}$ (red line), for the time-lapse data of the whole-wing captured at 5-min intervals starting from 15 h 30 min APF (**b**) and time-lapse data of the C region of the wing captured at 1-min intervals starting from 24 h APF (**d**). Blue line: The value obtained by using the velocity field measured using PIV for $\nabla \cdot {\varvec{v}}$. Green line: The value obtained by using the trace of the strain rate tensor ($\textrm{Tr}{\hat{\textbf{G}}}$) for $\nabla \cdot {\varvec{v}}$. These time series data denote the moving average values with a window of width 30 min (**b**) and 3 min (**d**). **c**, **e** Comparison of Eq. 8 using different measurement methods of $\nabla \cdot {\varvec{v}}$. The analysis results are shown using the velocity field measured by PIV (red dots) and the trace of the strain rate tensor ($\textrm{Tr}{\hat{\textbf{G}}}$, blue dots). Each point represents data from an individual ROI. Data analyzed in (**b**) and (**d**) are used in (**c**) and (**e**), respectively

In Eq. 8, $\textrm{Tr}  \textbf{D}_\textrm{T} = \textrm{Tr}  \textbf{R} + \textrm{Tr}  \textbf{D} + \textrm{Tr}  \textbf{A}$ represents the change in the cell number density, with **R**, **D**, and **A** denoting the strains associated with cell rearrangement, division, and apoptosis, respectively. Given that cell rearrangement does not alter tissue area, $\textrm{Tr}  \textbf{R}$ is expected to be zero (Eq. 9), whereas cell division and apoptosis contribute to the area expansion ($\textrm{Tr}  \textbf{D} \ge 0$) and contraction ($\textrm{Tr}  \textbf{A} \le 0$), respectively. We examined whether the data from the wings exhibited the anticipated features (Fig. 4a). Our analysis of wing data revealed that $\textrm{Tr}\hat{\textbf{R}}$ was significantly smaller than the other components throughout the time course of wing development, supporting the relationship $\textrm{Tr}  \textbf{R} = 0$ (Eq. 9). Furthermore, $\textrm{Tr}  \textbf{D}$ was consistently positive, as expected, whereas $\textrm{Tr}\hat{\textbf{A}}$ was nearly zero ($\sim 1.5 \times 10^{-6}\mathrm{h^{-1}}$), indicating that apoptosis contributes minimally to deformation of the pupal wing. These results are consistent with the low frequency of apoptosis reported in the wing [14]. The findings serve as a foundation for validating the cell number density equation outlined below.

Next, we evaluated the cell number density equation (Eq. 8) by employing different measurement methods for tissue deformation and assessing the consistency among them. Specifically, the left-hand side of Eq. 8 was evaluated as follows: We separated the left-hand side as $\dot{\rho }+\rho \nabla \cdot {\varvec{v}}$, where $\dot{\rho } \equiv \partial _t\rho + \varvec{v}\cdot \nabla \rho$. The time derivatives for $\hat{\textbf{M}}^{(1)}$, and consequently for the density $\hat{\rho } = 1/\pi |\hat{\textbf{M}}^{(1)}|^{1/2}$, were calculated as the difference between two consecutive time frames. In this way, we evaluated the Lagrange derivative $\dot{\rho }$ (but not $\partial _t \rho$), since the variables were measured using ROIs defined by tracking individual cells, as described in the Materials and Methods Sect. 3.8. Furthermore, the divergence of the velocity field, $\nabla \cdot \varvec{v}$, can be quantified in two ways; one using the total tissue strain rate $\textbf{G}$ obtained using the texture tensor through $\nabla \cdot \varvec{v} = \textrm{Tr} \textbf{G}$, and the other by direct velocity field measurement using PIV. The right-hand side $\textrm{Tr}  \textbf{D}_\textrm{T}$ was quantified by summing the respective contributions from morphogenetic cell events, specifically $\textrm{Tr}\hat{\textbf{R}}$, $\textrm{Tr}\hat{\textbf{D}}$, and $\textrm{Tr}\hat{\textbf{A}}$. Collectively, there are three ways to calculate the temporal evolution of the cell number density: $\textrm{Tr}{\hat{\textbf{G}}}$ and $\hat{\textbf{M}}^{(1)}$, PIV and $\hat{\textbf{M}}^{(1)}$, and the sum of the decomposed $\textrm{Tr}  \textbf{D}_\textrm{T}$. The time series data obtained from these measurements were plotted, with averages presented for the whole-wing (Fig. 4b) and the C region of the wing (Fig. 4d). The results from the two measurements of the velocity divergence $\nabla \cdot \varvec{v}$ were almost identical (blue and red lines in Figs. 4b, d and blue and red dots in Fig. 4c, e), indicating that measuring the velocity field $\varvec{v}$ by PIV can effectively be replaced by the texture tensor method with high accuracy, consistently with the results in Sect. 4.3. More importantly, all three time series closely match each other, providing strong support for the cell number density equation (Eq. 8).

### Evaluation of assignment rules of the strain rate decomposition

In the texture analysis, half-links that disappear and newly appear between two consecutive time frames are linked to specific morphogenetic cell events (Eq. 5; Fig. 1b). This assignment rule is not unique; for example, the approach proposed in Ref. [14] attributed the half-link from adjacent cells to a divided cell-to-cell division, it can also be assigned to a rearrangement (Supporting Information B and Fig. S5). To assess the impact of different assignment rules of the strain rate decomposition on the consistency of the time evolution equation for the cell number density Eq. 8, we conducted an evaluation (Supporting Information B for details). Our analysis revealed that alternative rules led to significant discrepancies with the cell number density equation, as shown in equation (Fig. S5). This suggests that the assignment rule proposed in Ref. [14] offered a consistent and optimal decomposition of the tissue strain to morphogenetic cell events.

### Assessment of the kinematic equations

**Fig. 5:**
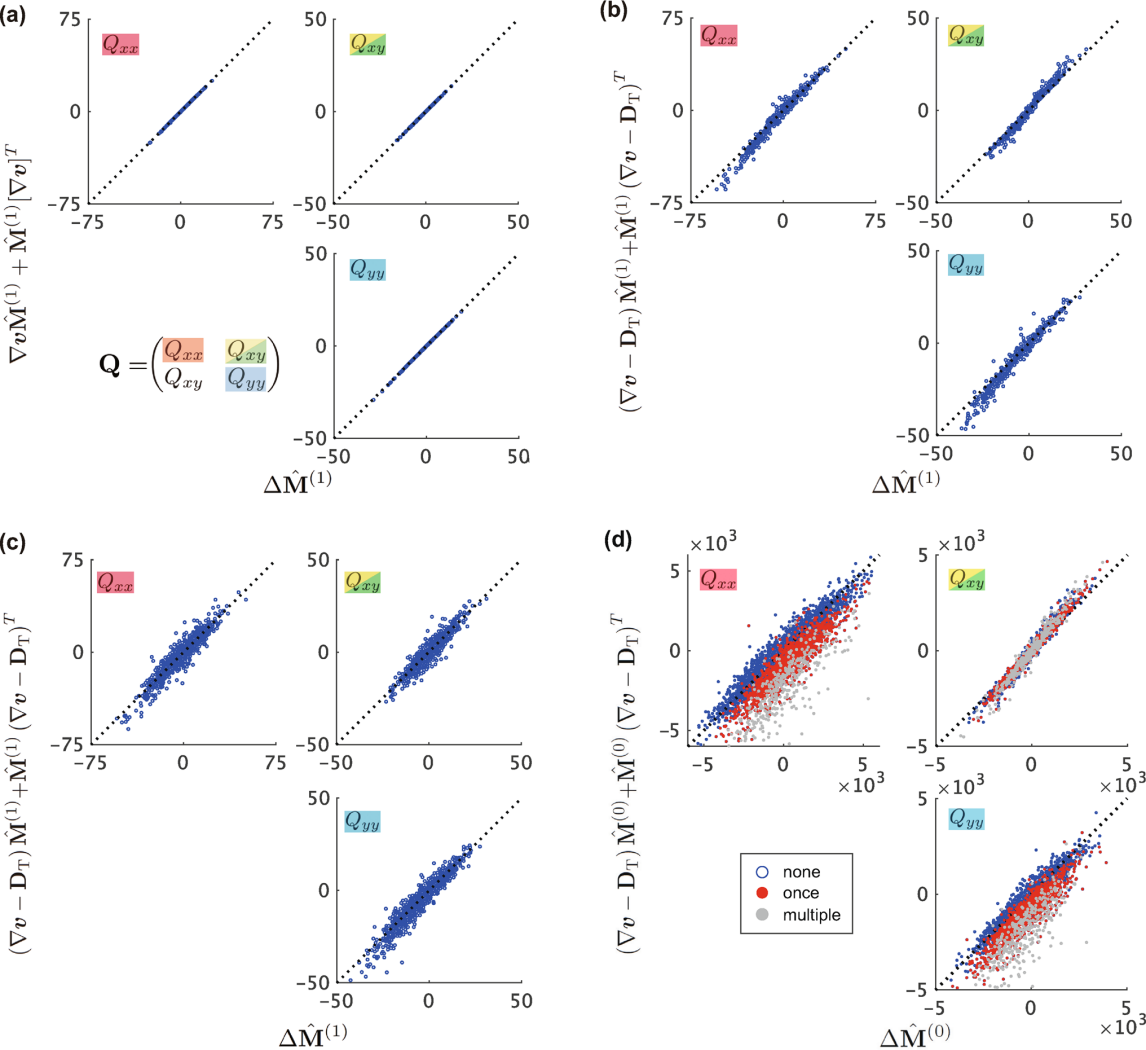
Accuracy of the kinematic equation for different definitions of $\hat{\textbf{M}}$. **a**–**d** The components of each tensor were evaluated for $\hat{\textbf{M}}^{(1)}$ (**a**–**c**) and $\hat{\textbf{M}}^{(0)}$ (**d**) using strain tensors based on $\hat{\textbf{F}}$ (**a**, **b**, **d**) and PIV-measured $\nabla \varvec{v}$ (**c**). Each point represents data from an individual ROI. In (**a**), only data from ROIs without topological deformations were plotted. In (**d**), point colors indicate the frequency of occurrence of cell division within the ROI: only once (red) or multiple times (gray) within the ROI

*Without topological deformation.* In the following analysis, we assess the kinematic relationship of $\textbf{M}$, represented in Eq. 7, where $\textbf{M}$ is replaced by the experimentally measured $\hat{\textbf{M}}^{(1)}$. First, we examined the scenario without topological deformation (i.e., $\textbf{D}_\textrm{T} = 0$). In the absence of topological deformation, the change in texture tensor $\hat{\textbf{M}}^{(1)}$ between consecutive time points obeys

16 $$\begin{aligned} \Delta \hat{\textbf{M}}^{(1)} = \left( \nabla \varvec{v} \hat{\textbf{M}}^{(1)} + \hat{\textbf{M}}^{(1)} [\nabla \varvec{v}]^T \right) \Delta t+ \mathcal {O}\left( \Delta t^2\right) \end{aligned}$$

where $\Delta t$ denotes the time interval between frames (Eq. S13 in Supporting Information A). By utilizing data from ROIs with no topological changes in the connection of half-links (*that is*, no cell rearrangement, division, or apoptosis; $\hat{\textbf{R}}=\hat{\textbf{D}} = \hat{\textbf{A}} = 0$), we evaluated the correspondence between the left- and right-hand sides of Eq. 16. Neglecting the higher-order terms of $\mathcal {O}(\Delta t^2)$, we observed an excellent agreement (Fig. 5a and Fig. S6). The $\mathcal {O}(\Delta t^2)$ term was sufficiently small, with a relative magnitude of approximately $1.2\times 10^{-2}$, considered negligible. These findings provide support for the kinematic equation, Eq. 7, in cases without topological deformation.

The original definition of the texture tensor, $\textbf{M}^{(0)}$, as well as the alternative definitions $\textbf{M}^{(2)}$, $\textbf{M}^{(3)}$, and $\textbf{M}^{(4)}$ (Supporting Information C), yielded excellent agreement between the left- and right-hand sides of Eq. 16 (Fig. S7). This is because each term of ${\varvec{l}}_{ik}\otimes {\varvec{l}}_{ik}$ satisfied the kinematic equations and Eq. 7 remained unchanged up to a scaling factor of $\hat{\textbf{M}}$.

*With topological deformation.* Next, we performed the same analysis using ROI data involving topological deformation. The kinematic equation for $\textbf{M}$, Eq. 7, was satisfied with minor deviations when the modified texture tensor $\hat{\textbf{M}}^{(1)}$ was employed (Fig. 5b, c and Fig. S8). This consistency was observed regardless of whether the strain rate tensors were determined using $\hat{\textbf{F}}$ or PIV-measured $\nabla \varvec{v}$.

In sharp contrast, the original texture tensor $\hat{\textbf{M}}^{(0)}$ failed to satisfy the kinematic equation (Fig. 5d and Fig. S9). The degree of deviation from the identity line was notably influenced by the number of cell divisions (blue, red, and gray points in Fig. 5d indicate the data from ROIs with zero, one, and more than two divisions, respectively). This suggests that the deviation stemmed from a lack of proper normalization by the half-links $N_\textrm{h}$ (Eq. 10).

Alternative definitions of the texture tensors $\hat{\textbf{M}}^{(2)}$ and $\hat{\textbf{M}}^{(3)}$ also resulted in larger deviations from the identity line compared with those obtained using $\hat{\textbf{M}}^{(1)}$ (Fig. S10a and b), which can be attributed to improper normalization. The appropriate normalization factor for representing the cell shape is the half-link number, $N_\textrm{h}$, rather than the cell number, $N_\textrm{c}$. $\hat{\textbf{M}}^{(4)}$ is in good agreement with Eq. 7 (Fig. S10c), comparable to the result obtained using $\hat{\textbf{M}}^{(1)}$. This is because the normalization factor $\sim N_\textrm{c} \sum _{i}n_i$ in the definition of $\hat{\textbf{M}}^{(4)}$ (Eq. S23) is of the same order as $N_\textrm{h}$. These findings underscore the robustness of quantification when the texture tensor utilized for measurement is appropriately normalized. Overall, our data demonstrated that a properly normalized texture tensor, $\hat{\textbf{M}}^{(1)}$, yielded quantitative data on tissue deformation that was highly consistent with the kinematic equations proposed in Ref. [19].

### Reproducibility of the analysis among samples

**Fig. 6:**
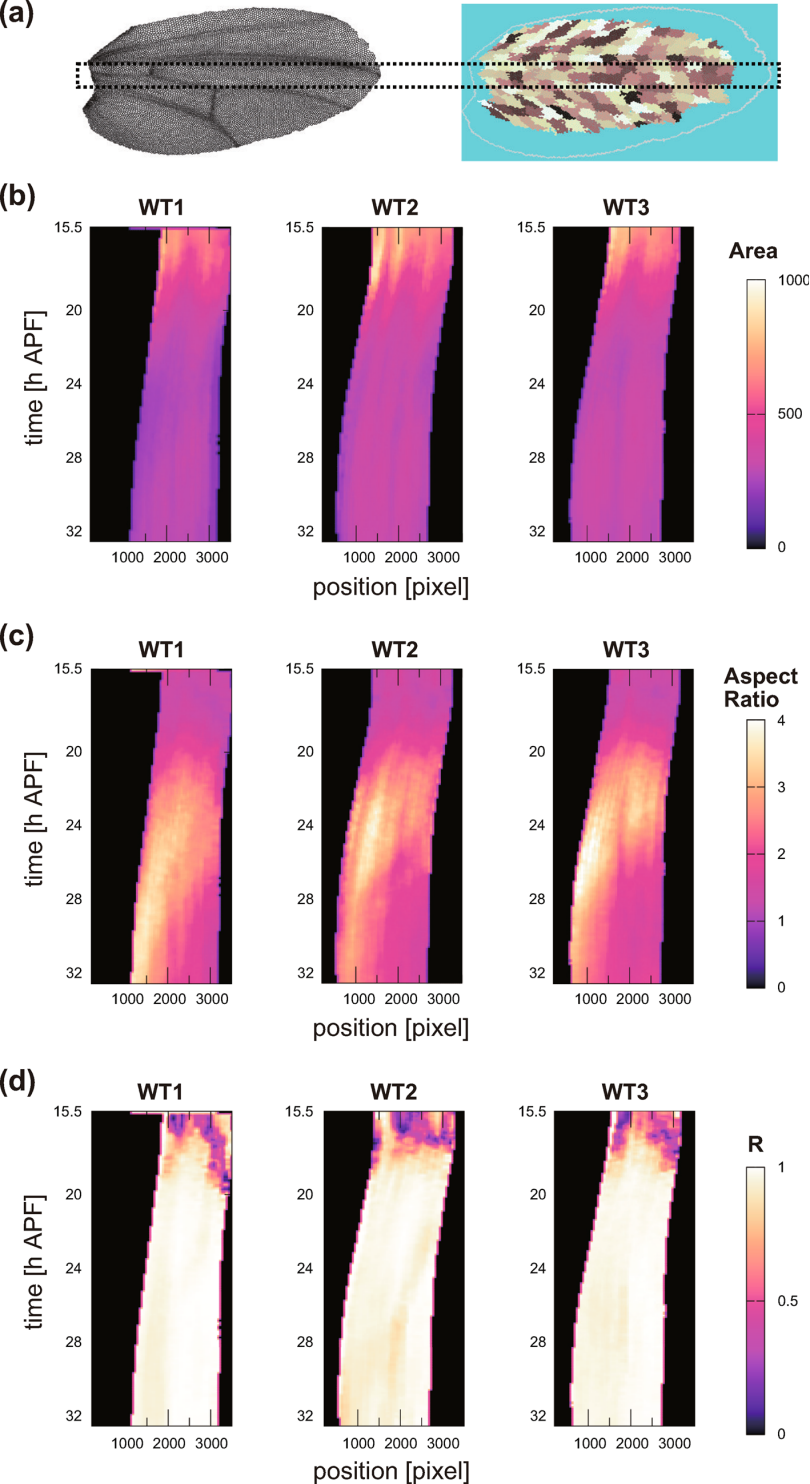
Reproducibility of cell shape quantities analyzed using the texture tensor across three wings samples. **a** Analyzed area of *Drosophila* wing (the area surrounded by the black dotted line). The area was set to include the C region. **b**–**d** Kymographs of cell shape quantities derived from the eigenvalues ($\lambda _1, \lambda _2$) and eigenvectors of the texture tensor $\hat{\textbf{M}}^{(1)}$. Quantities were calculated as moving average over a 200-px width (denoted by angle brackets) and are displayed at 50-px intervals. **b** Cell area, $\langle \pi \sqrt{\lambda _1\lambda _2} \rangle$. **c** Aspect ratio, $\langle \sqrt{\lambda _1/\lambda _2} \rangle$. **d** Local deviation in direction of cell elongation, calculated by $\left| \langle e^{i2\theta }\rangle \right|$ where $\theta$ denotes the angle of the eigenvector associated with $\lambda _1$

To ensure the reproducibility of the aforementioned results, we replicated the measurements in two additional samples of the whole-wings and compared the outcomes across all three samples. First, we evaluated the consistency of results obtained using the texture tensor $\hat{\textbf{M}}^{(1)}$. Kymographs of the cell shape characteristics during wing development in the region indicated in Fig. 6a are shown in Fig. 6b-d. The cell shape characteristics were derived from the texture tensor $\hat{\textbf{M}}^{(1)}$, and we calculated the eigenvalues of $\hat{\textbf{M}}^{(1)}$, $\lambda _1, \lambda _2$, and indicated the cellular area $\pi \sqrt{\lambda _1\lambda _2}$ (Fig. 6b), as well as the cell aspect ratio $\sqrt{\lambda _1/\lambda _2}$ (Fig. 6c) and the local deviation in the direction of cell elongation (direction of the eigenvector for $\lambda _1$) (Fig. 6d) [45]. These measurements exhibit spatial and temporal coherence across samples, validating the applicability of $\hat{\textbf{M}}^{(1)}$.

**Fig. 7:**
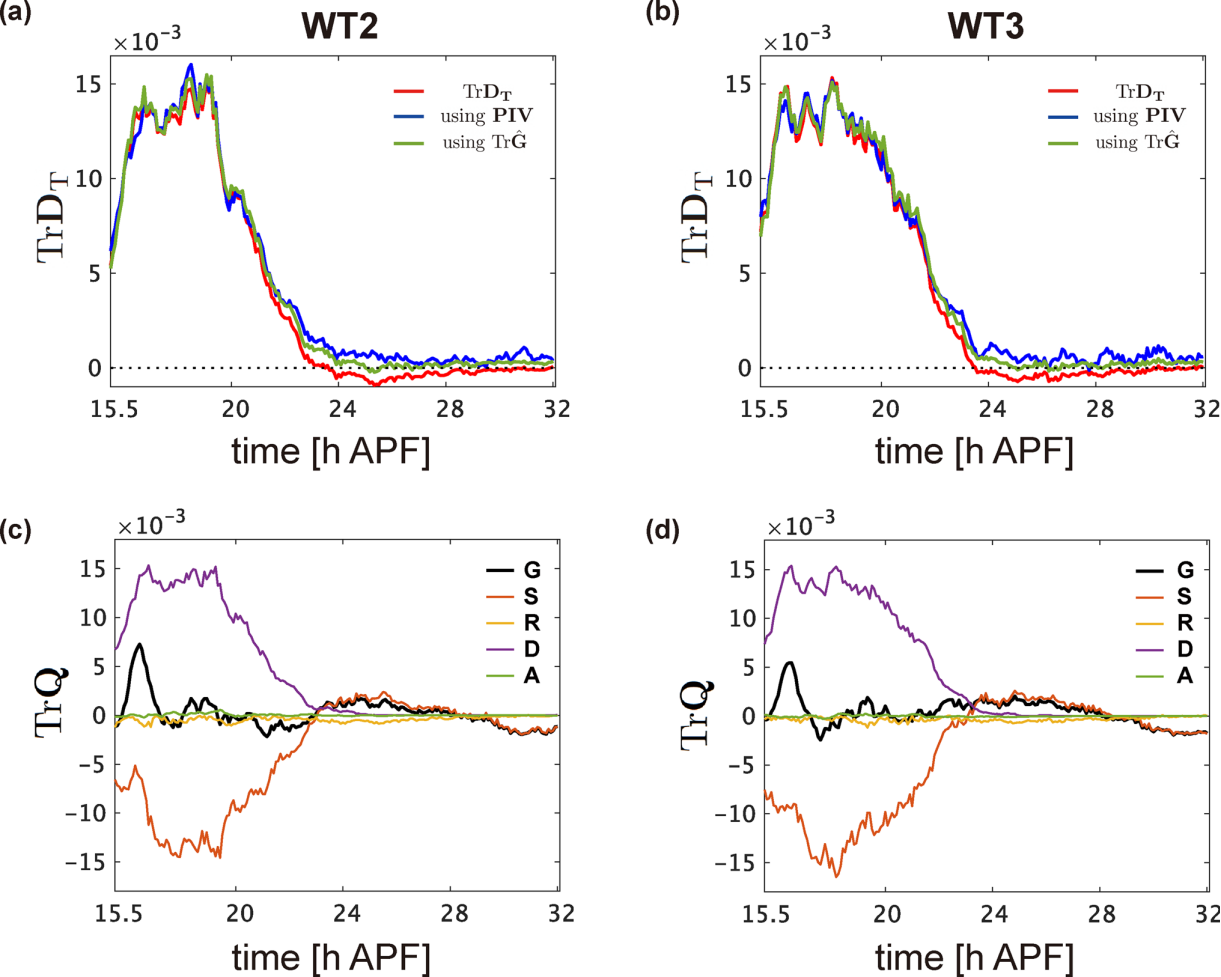
Reproducibility in validating the cell number density equations. **a**, **b** Time series data indicating the value obtained by dividing the left-hand side of Eq. 8 by $\hat{\rho }$ (blue and green lines), compared with $\textrm{Tr}  \textbf{D}_\textrm{T}$ (red line), for time-lapse data of the whole-wing captured at 5-min intervals starting from 15 h 30 min APF. **c**, **d** Time series of the traces of $\textrm{Tr}{\hat{\textbf{G}}}$ (black), $\textrm{Tr}{\hat{\textbf{S}}}$ (red), $\textrm{Tr}{\hat{\textbf{R}}}$ (yellow), $\textrm{Tr}{\hat{\textbf{D}}}$ (purple), and $\textrm{Tr}{\hat{\textbf{A}}}$ (green) for the same datasets as in (**a**, **b**). Data are from WT2 (**a**, **c**) and WT3 (**b**, **d**). Sample IDs are similar to those in Fig. 6

Next, we validated the strain rates tensors derived from $\hat{\textbf{M}}^{(1)}$. As previously mentioned, Figs. 3, S3, and S4 demonstrated consistency between the two ways of total strain rates measurements across different samples. Figure 7a and b is times series of $\textrm{Tr}  \textbf{D}_\textrm{T}$ in the other samples, both of which are similar to Fig. 4b. Trace components of the quantified strain tensors $\hat{\textbf{G}}$, $\hat{\textbf{S}}$, $\hat{\textbf{R}}$, $\hat{\textbf{D}}$, and $\hat{\textbf{A}}$ are also similar across different samples (Figs. 4a and 7c and d), supporting that the quantification procedure in our analysis is robust and reproducible.

## Discussion

Kinematic relationships play a key role in the dynamics of deforming materials, as they typically involve only a few parameters and represent the general and robust characteristics of a material compared to kinetics. In this study, we assessed the kinematic equations governing the cell shape tensor (Eq. 7) and cell number density (Eq. 8) using experimental data from *Drosophila* wings. To accomplish this, we introduced several potential definitions for the texture tensor, among which $\textbf{M}^{(1)}$ demonstrated a good agreement with direct pixel counting for cell area and with the second moment for cell shape. We also validated that the total strain tensor $\hat{\textbf{G}}$, derived from the change in $\textbf{M}^{(1)}$ between consecutive time frames, properly aligned with that obtained through PIV. By leveraging $\textbf{M}^{(1)}$, we demonstrated the compatibility of the kinematic equations proposed in Ref. [19] with experimental data, thereby validating the theoretical framework of the kinematic equations while also demonstrating the practical utility of the texture tensor $\textbf{M}^{(1)}$ as a reliable indicator for analyzing morphogenetic cell events.

Our analysis revealed that all definitions of the texture tensors, $\textbf{M}^{(m)}$ $(m=0,1,2,3,4)$, support the kinematic equation for $\textbf{M}$ (Eq. 7) in the absence of topological changes in the texture (Fig. 5a and Fig. S7). However, when topological changes in the texture were involved, only the analyses using $\textbf{M}^{(1)}$ and $\textbf{M}^{(4)}$ successfully supported the kinematic equation, whereas those using other definitions, including the original $\textbf{M}^{(0)}$, did not. This discrepancy is attributed to the selection of normalization factors. In the presence of topological changes, the number of half-links changes, necessitating an update to the normalization factors. The analysis using $\textbf{M}^{(4)}$ yielded results comparable with those using $\textbf{M}^{(1)}$ (Fig. S2d and Fig. S10c) owing to their normalization factors being of similar magnitude ($N_\textrm{h} \sim N_\textrm{c} \sum _{i}n_i$), rendering them invariant to the size of the ROI. This finding further emphasizes that the specific definition of the texture tensor is not crucial as long as an appropriate predetermined normalization factor is applied, hence underscoring the robustness of the analysis using the texture tensor.

The kinematic equation for $\textbf{M}$, Eq. 7, was derived assuming the epithelial tissue can be modeled as a tiling of polygons, where changes in cell shape are tightly coupled with tissue strains [18, 19]. In this equation, cell shape changes are assumed to be governed solely by tissue strains, $\nabla {\varvec{v}}$, and topological changes, $\textbf{D}_\textrm{T}$. For example, in the absence of topological changes ($\textbf{D}_\textrm{T}=0$), cell shape changes ($\dot{\textbf{M}}$) and total tissue strain ($\nabla {\varvec{v}}$) must remain compatible with each other. Note that this kinetic relationship differs from that of other systems such as liquid crystals [46], in which molecular alignment, represented by the nematic tensor, can evolve independently of the macroscopic velocity, as indicated by appearance of a relaxation term in the time evolution equation for the nematic tensor. Furthermore, the cell number density equation Eq. 8 is directly derived from the kinematic equation Eq. 7 as discussed in Sect. 2.2. Our data analysis using the revised texture tensor method confirmed that both equations (Sects. 4.4 and 4.6) are highly consistent with quantitative data of tissue deformation and morphogenetic cell events.

The current texture tensor analysis procedure for decomposing strain rates, as described in [14] and Supporting Information A, has room for further improvement. Specifically, the method does not account for rotational deformation, and the choice of normalization in deriving Eq. S14, as well as the definition of the residual term $\Psi$ in Eq. S17, lacks direct interpretability in terms of cellular or continuum deformation kinematics. While a deeper exploration of the theoretical framework lies beyond the scope of the present study, it represents an important and intriguing avenue for future research. We also note that in situations where cell–cell connections are weaker or looser, such as in mesenchymal tissues, modifications to both the kinematic equations and the corresponding strain measurement methods may be required.

Having established the kinematics, kinetic relationship should be addressed. However, validating kinetics requires more advanced techniques, as it involves numerous parameters in constitutive equations that, unlike kinematics addressed in this study, may not be directly measurable. One promising approach is data assimilation, a statistical technique that integrates computational model with observed data [47]. By iteratively combining simulation and fitting, data assimilation enables not only quantitative, data-driven simulation but also system identification, including the evaluation of model parameters, their uncertainties, and the goodness-of-fit of the model [48, 49]. This method has been successfully applied to biological systems [50–53]. The precision in the kinematic relationships achieved through our texture tensor analysis provides a solid foundation for such an approach. However, implementing and testing it requires substantial effort and will be the focus of future studies.

In conclusion, we cross-validated the kinematic equations of multi-scale continuum model and the strain measurement methods for epithelial tissue. The approach used in this study can be useful for testing other related methods that employ different kinematic and kinetic frameworks. We anticipate that the quantitative comparison and validation of our methods, in conjunction with related approaches, will aid in bridging tissue- and cell-scales in epithelial morphogenesis.

## Supplementary Information

Below is the link to the electronic supplementary material.

Supplementary file 1 (pdf 19085 KB)

## Data Availability

The data are available from the lead contact upon reasonable request. The code for texture analysis can be downloaded from https://github.com/IshiharaLab/TextureTensorAnalysis.
