# Supplementary material for: In vivo assessment of kinematic relationships for epithelial morphogenesis
Source: Eur Phys J E Soft Matter. 2025 Jun 15;48(6-7):31. doi: 10.1140/epje/s10189-025-00495-2 (PMC12167722; doi:10.1140/epje/s10189-025-00495-2)
Supplement: Supplementary file 1 — (pdf 19085 KB) [file 10189_2025_495_MOESM1_ESM.pdf]

Supporting Information

A Tissue deformation analysis by texture tensors

We summarize the texture tensor analysis utilized in the main text to quantify cell and tissue deformations. This method involves calculating strain tensors that result from morphogenetic cell events by examining temporal changes in the texture tensor $\hat{\mathbf{M}}^{(0)}$ (Eq. 10 of the main text). While our approach is based on the method outlined by Guirao et al. [1], we offer an alternative derivation of the deformation gradient tensor, $\hat{\mathbf{F}}$ (Sect. A2). Furthermore, the specific expression of the strains to be measured differs slightly from the previous ones, with the deviation being of the order $\mathcal{O}(\Delta t^2)$ (Sect. A3).

The data analysis workflow is summarized in Fig. S1. In this study, coarse-grained measurement were performed using ROIs defined by cell-tracking data (*i.e.*, the same cells are tracked from the initial to the final time points; Sect. 3.7 and Sect. 3.8 in the main text). This allows for evaluation of temporal changes in the cell shape field $\hat{\mathbf{M}}$ without accounting for influx and efflux, in other words, we evaluate the Lagrange derivative of $\hat{\mathbf{M}}$ at each time point.

A1 Temporal changes in the texture tensor

The change in $\hat{\mathbf{M}}^{(0)}$ between two consecutive time frames is defined as follows:

$$\Delta\hat{\mathbf{M}} = \hat{\mathbf{m}} - \hat{\mathbf{M}} = \sum_{n_\mathrm{h}} \frac{1}{2}\omega \boldsymbol{l} \otimes \boldsymbol{l} - \sum_{N_\mathrm{h}} \frac{1}{2} W \boldsymbol{L} \otimes \boldsymbol{L}. \tag{S1}$$

In this expression, uppercase and lowercase letters represent quantities measured at the earlier and later time points of the consecutive frames, respectively (*i.e.*, at time t and $t + \Delta t$). With the cell-tracking data, $\hat{\mathbf{m}}$ and $\hat{\mathbf{M}}$ are calculated from ROIs composed of the same cells, or their mother or daughter cells (Sect. 3.7 in the main text; Fig. S1c, d). Thus, $\Delta\hat{\mathbf{M}}/\Delta t$ evaluates Lagrange derivatives $\dot{\hat{\mathbf{M}}} \equiv \partial_t \hat{\mathbf{M}} + \boldsymbol{v} \cdot \nabla \hat{\mathbf{M}}$ at time point t (Sect. 3.8). At time $t + \Delta t$, the total number of half-links n_h is the sum of the number of conserved links, n_c, and the number of links that appeared, n_a, between the time frames. Similarly, at time t, the total number of half-links N_h is the sum of the number of conserved links, $N_\mathrm{c} = n_\mathrm{c}$, and the number of links that disappeared, N_d. The decomposition of Eq. S1 is as follows:

$$\begin{aligned} \Delta\hat{\mathbf{M}} &= \left(\sum_{n_\mathrm{c}} \frac{1}{2}\omega_\mathrm{c} \boldsymbol{l}_\mathrm{c} \otimes \boldsymbol{l}_\mathrm{c} - \sum_{N_\mathrm{c}} \frac{1}{2} W_\mathrm{c} \boldsymbol{L}_\mathrm{c} \otimes \boldsymbol{L}_\mathrm{c} \right) - \left(\sum_{n_\mathrm{a}} \frac{1}{2}\omega_\mathrm{a} \boldsymbol{l}_\mathrm{a} \otimes \boldsymbol{l}_\mathrm{a} - \sum_{N_\mathrm{d}} \frac{1}{2} W_\mathrm{d} \boldsymbol{L}_\mathrm{d} \otimes \boldsymbol{L}_\mathrm{d} \right) \\ &= \overline{\mathbf{C}} + \overline{\mathbf{T}} \; . \end{aligned} \tag{S2}$$

The first term enclosed in brackets in Eq. S2 comprises links that maintain their neighboring relationships, denoted as $\overline{\mathbf{C}}$. The second term, $\overline{\mathbf{T}}$, signifies the overall change attributed to the topological processes and can be decomposed as $\overline{\mathbf{T}} = \sum_\mathrm{P} \overline{\mathbf{P}} = \overline{\mathbf{R}} + \overline{\mathbf{D}} + \overline{\mathbf{A}} \; \left(+\overline{\mathbf{J}}\right)$, where $\overline{\mathbf{R}}$, $\overline{\mathbf{D}}$, $\overline{\mathbf{A}}$, and $\overline{\mathbf{J}}$ indicate that rearrangement, division, ap-

optosis, and flux ($\overline{\mathbf{J}}$ appeared only in the Eulerian description and is absent in our analysis). The abbreviations $\overline{\mathbf{C}} = \hat{\mathbf{m}}_{\rm c} - \hat{\mathbf{M}}_{\rm c}$ and $\overline{\mathbf{T}} = \hat{\mathbf{m}}_{\rm a} - \hat{\mathbf{M}}_{\rm d}$ are utilized. These tensors have squared length dimensions.

The decomposition of $\overline{\mathbf{T}}$ into $\overline{\mathbf{R}}$, $\overline{\mathbf{D}}$, and $\overline{\mathbf{A}}$ was performed as follows: $\overline{\mathbf{T}}$ was calculated from the links that disappeared or appeared between consecutive frames. As explained in the main text, each link comprises two half-links $\boldsymbol{l}_{ik}$ and $\boldsymbol{l}_{ki}(= -\boldsymbol{l}_{ik})$ (or $\boldsymbol{L}_{ik}$ and $\boldsymbol{L}_{ki} = -\boldsymbol{L}_{ik}$), with both half-links belonging to the same link being assigned to the same morphogenetic cell event. The allocation of half-links to division and apoptosis took precedence over rearrangements. An example of the assignment is shown in Fig. 1b. For further discussion on this topic, refer to Sect. 4.5 in the main text and Sect. B in the Supporting Information.

A2 Deformation gradient tensor F for tissue deformation

Deformation of a continuum material can be described using a deformation gradient tensor $\mathbf{F}$ [2]. In our texture tensor analysis, we calculate the empirical deformation gradient tensor $\hat{\mathbf{F}}$ based on the half-links obtained through *in vivo* measurements. Consider the deformation of a continuum object, where a material point $\boldsymbol{r}$ at time t is mapped to $\boldsymbol{R} = \boldsymbol{r} + \boldsymbol{u}$ at time $t + \Delta t$, with $\boldsymbol{u}$ representing the displacement vector. The relative position $d\boldsymbol{r}$ between two points at infinitesimal distances changes to $d\boldsymbol{R} = \mathbf{F}d\boldsymbol{r}$ owing to deformation, where $\mathbf{F}$ is the deformation gradient tensor defined as

$$\mathbf{F} \equiv \frac{\partial \boldsymbol{R}}{\partial \boldsymbol{r}} = \mathbf{I} + \nabla \boldsymbol{u} \tag{S3}$$

with the identity matrix $\mathbf{I}$.

In practice, $\mathbf{F}$ is evaluated using half-links contained in the corresponding ROI between consecutive time frames. When the tissue deforms without topological changes ($\overline{\mathbf{T}} = 0$), all links are conserved between two consecutive time frames. In such cases, a conserved link changes from $\boldsymbol{L}_{\rm c}$ to $\boldsymbol{l}_{\rm c}$, satisfying $\boldsymbol{l}_{\rm c} = \mathbf{F}\boldsymbol{L}_{\rm c}$. Even in cases involving topological changes ($\overline{\mathbf{T}} \neq 0$), we can assume that most links are conserved, with only a small fraction of half-links appearing and disappearing. Assuming affine deformation in each ROI and a constant $\mathbf{F}$, $\mathbf{F}$ is determined from the experimental data by minimizing the function

$$\Phi_0(\hat{\mathbf{F}}) = \sum_i^{N_{\rm c}} \omega_i |\boldsymbol{l}_i - \hat{\mathbf{F}}\boldsymbol{L}_i|^2 \ . \tag{S4}$$

where the summation is performed over the conserved links. $\mathbf{F}$ is estimated as follows:

$$\mathbf{F} = \hat{\mathbf{F}}_0 \equiv \langle \boldsymbol{l} \otimes \boldsymbol{L} \rangle_{\rm c} \langle \boldsymbol{L} \otimes \boldsymbol{L} \rangle_{\rm c}^{-1} \tag{S5}$$

where $2{\times}2$ tensor $\langle \boldsymbol{l} \otimes \boldsymbol{L} \rangle_{\rm c}$ is defined by $\langle \boldsymbol{l} \otimes \boldsymbol{L} \rangle_{\rm c} \equiv \sum_i^{N_{\rm c}} \omega_i \boldsymbol{l}_i \otimes \boldsymbol{L}_i$, and $\langle \boldsymbol{L} \otimes \boldsymbol{L} \rangle_{\rm c}$ is defined

similarly. Furthermore, $\mathbf{F}$ can be determined by considering an alternative function[1];

$$\Phi_1(\hat{\mathbf{F}}) = \sum_i^{N_c} W_i |\hat{\mathbf{F}}^{-1}\boldsymbol{l}_i - \boldsymbol{L}_i|^2 \tag{S6}$$

and $\Phi_1(\hat{\mathbf{F}})$ is minimal at

$$\mathbf{F} = \hat{\mathbf{F}}_1 \equiv \langle \boldsymbol{l} \otimes \boldsymbol{l} \rangle_c \langle \boldsymbol{L} \otimes \boldsymbol{\ell} \rangle_c^{-1} \ . \tag{S7}$$

Notably, $\hat{\mathbf{F}}_0$ and $\hat{\mathbf{F}}_1$ satisfy the following relationships:

$$\hat{\mathbf{m}}_c = \hat{\mathbf{F}}_1 \hat{\mathbf{M}}_c \hat{\mathbf{F}}_0^T \ . \tag{S8}$$

Furthermore, because $\hat{\mathbf{m}}_c$ and $\hat{\mathbf{M}}_c$ are both symmetric tensors, *that is*, $\hat{\mathbf{m}}_c = \hat{\mathbf{m}}_c^T = \hat{\mathbf{F}}_0 \hat{\mathbf{M}}_c \hat{\mathbf{F}}_1^T$, $\hat{\mathbf{F}}_0$ and $\hat{\mathbf{F}}_1$ satisfy $\hat{\mathbf{F}}_1 \hat{\mathbf{M}}_c \hat{\mathbf{F}}_0^T = \hat{\mathbf{F}}_0 \hat{\mathbf{M}}_c \hat{\mathbf{F}}_1$.

$\hat{\mathbf{F}}_0$ and $\hat{\mathbf{F}}_1$ represent two empirical approximations of the deformation gradient tensor $\mathbf{F}$, anticipated to have a similar construction. Moreover, they should align in the case of the ideal pure affine deformation, resulting in the following relationship:

$$\hat{\mathbf{m}}_c = \mathbf{F} \hat{\mathbf{M}}_c \mathbf{F}^T \ . \tag{S9}$$

We chose the deformation gradient tensor as the arithmetic mean in our analysis.

$$\hat{\mathbf{F}} \equiv \frac{1}{2}\left(\hat{\mathbf{F}}_0 + \hat{\mathbf{F}}_1\right) \ . \tag{S10}$$

This quantity, calculated from conserved half-links in the ROI, was utilized for the analysis discussed in the main text.

We evaluated the relative mismatch using data from *Drosophila* epithelial tissues (pupal wing and notum).

$$\delta = \frac{||\hat{\mathbf{m}}_c - \hat{\mathbf{F}} \hat{\mathbf{M}}_c \hat{\mathbf{F}}^T||}{||\overline{\mathbf{C}}||} \ , \tag{S11}$$

where the norm of the second-order tensor $\mathbf{a}$ is defined as $||\mathbf{a}||^2 \equiv \sum_{ij} a_{ij}^2$. The mismatch δ is sufficiently small ($< 4.0 \times 10^{-3}$), validating the appropriateness of $\hat{\mathbf{F}}$ as the definition of the deformation gradient tensor. Moreover, we examined the mismatch using the geometric means of $\hat{\mathbf{F}}_0$ and $\hat{\mathbf{F}}_1$, $\hat{\mathbf{F}}_g \equiv \left(||\hat{\mathbf{F}}_1||/||\hat{\mathbf{F}}_0||\right)^{1/2} \hat{\mathbf{F}}_0$, instead of $\hat{\mathbf{F}}$. The small mismatch ($< 8.0 \times 10^{-3}$) between $\hat{\mathbf{F}}_0$ and $\hat{\mathbf{F}}_1$ suggests that they are similar, and our analysis results are not significantly influenced by the choice of their means. The symmetric part of the total strain-rate tensor is calculated using $\hat{\mathbf{F}}$ as follows:

$$\hat{\mathbf{G}} = \frac{1}{2\Delta t}\left(\hat{\mathbf{F}}^T \hat{\mathbf{F}} - \mathbf{I}\right) , \tag{S12}$$

which was utilized in our analysis (Eqs. 2, 4, and 11 in the main text).

---

[1] $\Phi_1(\mathbf{F})$ expressed as $\Phi_1(\mathbf{F}) = \sum_i^{N_c} W_i \left(\boldsymbol{l}_i - \mathbf{F}\boldsymbol{L}\right)^T \mathbf{B}^{-1} \left(\boldsymbol{l}_i - \mathbf{F}\boldsymbol{L}\right)$, using the left Cauchy-Green tensor $\mathbf{B} \equiv \mathbf{F}\mathbf{F}^T$. In studies on continuum mechanics, $\mathbf{B}^{-1}$ is interpreted as the metric tensor of the $\boldsymbol{x}$-space [3].

For deformations without topological processes, $\hat{\mathbf{m}}_c = \hat{\mathbf{m}}$ and $\hat{\mathbf{M}}_c = \hat{\mathbf{M}}$ applies. Eqs. S3 and S9 lead to $\hat{\mathbf{m}} = (\mathbf{I} + \nabla \boldsymbol{u})\, \hat{\mathbf{M}}\, (\mathbf{I} + \nabla \boldsymbol{u})^T$, and then $\Delta\hat{\mathbf{M}} = \hat{\mathbf{m}} - \hat{\mathbf{M}}$(Eq. S1) reads

$$\Delta\hat{\mathbf{M}} = (\nabla \boldsymbol{u})\hat{\mathbf{M}} + \hat{\mathbf{M}}(\nabla \boldsymbol{u})^T + \nabla \boldsymbol{u}\hat{\mathbf{M}}(\nabla \boldsymbol{u})^T \; . \tag{S13}$$

The equation obtained by omitting the third term on the right-hand side (higher-order term with respect to Δt) corresponds to Eq. 16 in the main text.

A3 Dimensionless symmetric strain rate tensors

The strain rate tensors $\mathbf{G}$, $\mathbf{S}$, $\mathbf{R}$, $\mathbf{D}$, and $\mathbf{A}$ utilized in the continuum theory have units of the inverse of time. We outline the calculation of the corresponding empirical strain tensors $\hat{\mathbf{G}}$, $\hat{\mathbf{S}}$, $\hat{\mathbf{R}}$, $\hat{\mathbf{D}}$, and $\hat{\mathbf{A}}$ from experimental data. Assuming the preservation of most links, the conserved half-links are utilized to derive the deformation gradient with $\hat{\mathbf{M}}_c$ as the reference state. The dimensionless symmetric tensors for $\mathbf{Q} \in \overline{\mathbf{C}}, \overline{\mathbf{R}}, \overline{\mathbf{D}}, \overline{\mathbf{A}}$ are defined as follows:

$$\tilde{\mathbf{Q}} = \frac{1}{4}\left[\mathbf{Q}\hat{\mathbf{M}}_c^{-1} + \hat{\mathbf{M}}_c^{-1}\mathbf{Q}^T\right] \tag{S14}$$

where the tilde denotes an operation that produces dimensionless symmetric tensors using $\hat{\mathbf{M}}_c$. $\tilde{\overline{\mathbf{C}}}$ is calculated as

$$\begin{aligned}
\tilde{\overline{\mathbf{C}}} &= \frac{1}{4}\left[\overline{\mathbf{C}}\hat{\mathbf{M}}_c^{-1} + \hat{\mathbf{M}}_c^{-1}\overline{\mathbf{C}}^T\right] \\
&= \frac{1}{4}\left[(\hat{\mathbf{m}}_c - \hat{\mathbf{M}}_c)\hat{\mathbf{M}}_c^{-1} + \hat{\mathbf{M}}_c^{-1}(\hat{\mathbf{m}}_c - \hat{\mathbf{M}}_c)^T\right] \\
&= \frac{1}{4}\left[(\hat{\mathbf{F}}\hat{\mathbf{M}}_c\hat{\mathbf{F}}^T)\hat{\mathbf{M}}_c^{-1} + \hat{\mathbf{M}}_c^{-1}(\hat{\mathbf{F}}\hat{\mathbf{M}}_c\hat{\mathbf{F}}^T)^T - 2\mathbf{I}\right] \\
&= \frac{1}{2}\left(\hat{\mathbf{F}}^T\hat{\mathbf{F}} - \mathbf{I}\right) + \frac{1}{4}\left[\hat{\mathbf{F}}\hat{\mathbf{M}}_c\hat{\mathbf{F}}^T\hat{\mathbf{M}}_c^{-1} - \hat{\mathbf{F}}^T\hat{\mathbf{F}} + \hat{\mathbf{M}}_c^{-1}\hat{\mathbf{F}}\hat{\mathbf{M}}_c\hat{\mathbf{F}}^T - \hat{\mathbf{F}}^T\hat{\mathbf{F}}\right] \\
&= \hat{\mathbf{E}} + \frac{1}{4}\left[\hat{\mathbf{\Psi}}\hat{\mathbf{M}}_c^{-1} + \hat{\mathbf{M}}_c^{-1}\hat{\mathbf{\Psi}}^T\right] \\
&= \hat{\mathbf{E}} + \tilde{\mathbf{\Psi}}
\end{aligned} \tag{S15}$$

where we utilized

$$\hat{\mathbf{E}} \equiv \frac{1}{2}\left(\hat{\mathbf{F}}^T\hat{\mathbf{F}} - \mathbf{I}\right) \tag{S16}$$

$$\hat{\mathbf{\Psi}} \equiv \hat{\mathbf{F}}\hat{\mathbf{M}}_c\hat{\mathbf{F}}^T - \hat{\mathbf{F}}^T\hat{\mathbf{F}}\hat{\mathbf{M}}_c \; . \tag{S17}$$

$\hat{\mathbf{E}} = \hat{\mathbf{G}}\Delta t$ represents the Green-Lagrange strain tensor with respect to the deformation between two consecutive time frames [4]. $\hat{\mathbf{\Psi}}$ is expressed as $\hat{\mathbf{\Psi}} = \hat{\mathbf{m}}_c - \hat{\mathbf{F}}^T\hat{\mathbf{m}}_c\hat{\mathbf{F}}^{-T}$, and vanishes if $\hat{\mathbf{M}}_c$ and $\hat{\mathbf{F}}$ commute.

Ref. [1] adopted $\hat{\mathbf{E}}^* = \frac{1}{2}\left(\hat{\mathbf{F}}\hat{\mathbf{F}}^T - \mathbf{I}\right)$ as a measure of deformation instead of $\hat{\mathbf{E}}$. The difference, $\hat{\mathbf{E}} - \hat{\mathbf{E}}^* \simeq \mathcal{O}(\Delta t^2)$, is negligible (Fig. S11).

A4 Decomposition of the strain rate to cell morphogenetic events

The tissue strain tensor $\hat{\mathbf{G}}$ defined in Eq. S12 can be decomposed into the strains resulting from cell morphogenetic events. By substituting Eq. S15 into Eq. S2, we obtain

$$\hat{\mathbf{G}}\Delta t = \Delta\tilde{\mathbf{M}} - \tilde{\mathbf{\Psi}} - \sum_{\mathrm{P}} \tilde{\mathbf{P}} \, , \tag{S18}$$

where Eq. S14 was employed. Here, $\tilde{\mathbf{P}}$ represents the contribution from topological events, with each $\tilde{\mathbf{P}}$ calculated from the half-links assigned to the corresponding topological cellular event. $\Delta\tilde{\mathbf{M}}$ represents the total deformation of the ROI in terms of the size and shape, which is further partitioned into components that correspond to the respective cellular events. Notably, $N_{\mathrm{h}} = N_{\mathrm{c}} + N_{\mathrm{d}}$ and $n_{\mathrm{h}} = n_{\mathrm{c}} + n_{\mathrm{a}}$ represent the numbers of half-links at time t and $t + \Delta t$, respectively. N_{d} and n_{a} denote the numbers of disappearing and appearing half-links associated with topological cellular events, respectively. These are further decomposed into $N_{\mathrm{d}} = \sum_{\mathrm{P}} N_{\mathrm{P}}$ and $n_{\mathrm{a}} = \sum_{\mathrm{P}} n_{\mathrm{P}}$, where the subscript P denotes either T, D, or A. The change in the number of half-links is expressed as $\Delta N = n_{\mathrm{h}} - N_{\mathrm{h}} = n_{\mathrm{a}} - N_{\mathrm{d}} = \sum_{\mathrm{P}} (n_{\mathrm{P}} - N_{\mathrm{P}}) \equiv \sum_{\mathrm{P}} \Delta N_{\mathrm{P}}$. The deformation of the ROI from $\hat{\mathbf{M}}$ to $\hat{\mathbf{m}}$ is partitioned based on the numbers of half-links, as follows:

$$\Delta\tilde{\mathbf{M}} = \left(\frac{N_{\mathrm{h}}}{n_{\mathrm{h}}}\tilde{\mathbf{m}} - \tilde{\mathbf{M}}\right) + \sum_{\mathrm{P}} \frac{\Delta N_{\mathrm{P}}}{n_{\mathrm{h}}}\tilde{\mathbf{m}}. \tag{S19}$$

The magnitude of $\tilde{\mathbf{m}}$ is normalized by $N_{\mathrm{h}}/n_{\mathrm{h}}$, rendering it comparable with $\tilde{\mathbf{M}}$. The residual fraction of $\tilde{\mathbf{m}}$ in the last term is attributed to topological cellular processes. Notably, the value of ΔN_{P} is expected to be positive for division (D), negative for apoptosis (A), and $\Delta N_{\mathrm{P}} \simeq 0$ for rearrangement (R) (Fig. 4 for the experimental validation).

These arguments enable the decomposition of tissue deformation into contributions from each cellular event. From Eqs. S18 and S19, we obtain:

$$\hat{\mathbf{G}}\Delta t = \underbrace{\left(\frac{N}{n}\tilde{\mathbf{m}} - \tilde{\mathbf{M}} - \tilde{\mathbf{\Psi}}\right)}_{\hat{\mathbf{S}}} + \underbrace{\sum_{\mathrm{P}} \left(\frac{\Delta N_{\mathrm{P}}}{n}\tilde{\mathbf{m}} - \tilde{\mathbf{P}}\right)}_{\hat{\mathbf{T}}=\sum_{\mathrm{P}} \hat{\mathbf{P}}} = \hat{\mathbf{S}} + \hat{\mathbf{T}}. \tag{S20}$$

The notation $\mathbf{D}_{\mathrm{T}}$ utilized in the continuum model [5] corresponds to $\hat{\mathbf{T}} = \hat{\mathbf{R}} + \hat{\mathbf{D}} + \hat{\mathbf{A}}$.

B Inconsistencies in cell number density equations resulting from the inappropriate assignment rules

In the analysis of texture tensor, each half-link is associated with a specific cell morphogenetic event: cell shape change (S), rearrangement (R), division (D), or apoptosis (A) (Fig. 1a). However, determining the assignment rule can be complex, particularly when dealing with topological changes in half-link connections during R, D, and A events. Consider a scenario in which a cell divides, as shown in Figs. S5a. The dividing cell(s) and their first-neighbor non-dividing cells are colored in green and gray, respect-

ively. Cells are categorized based on changes in their relationships with neighboring cells. The centers of dividing cells are denoted by light green points. For first-neighbor non-dividing cells, the centers of cells with an increased number of adjacent cells are represented by blue points, whereas those without such changes are denoted by black points. The question arises: to which cell morphogenetic event should the half-link between dividing and non-dividing cells be assigned? In ref. [1] and the main text of this study, the half-links are considered undirected edges, with both half-links between the pairs of cells attributed to the same morphogenetic event. Alternatively, considering half-links as directed edges could lead to a rule dependent on direction.

We assessed whether the consistency of the time evolution equation for cell number density (Eq. 8 in the main text) was influenced by the different assignment rules of the strain-rate decomposition. The assignment rule for the half-links adopted in the main text is shown in Fig. S5b. We reproduced the results shown in Fig. 4b. The results of the time evolution in Eq. 8 are shown in Fig. S5c with direction-dependent assignment rules applied. These rules include: (i) Assigning all half-links from white-dot cells to cell division. (ii) Assigning half-links from blue to white-dot cells to cell division owing to the division of the opposing cell. (iii) Considering half-links from black to white-dot cells as cell shape changes reflecting the consistent relationship with the opposite cell. (iv) Classifying half-links between black-dot and blue-dot cells as cell shape changes. The application of these rules resulted in a time series of cell number density that did not align with those obtained by substituting the deformation field with data from PIV.

Furthermore, we explored a scenario in which the assignment is independent of the direction of the half-links; however, the rules differ from those utilized by Guirao et al. [1]. The following assignment rules were employed: (i) Links between (white, white) and (white, blue)cells were assigned to cell division. (ii) Links between (black, white) and (black, blue)cells were assigned to cell shape changes. As shown in Fig. S5d, these rules resulted in a greater discrepancy compared with that observed in Fig. S5c, likely resulting from the underestimation of strain from topological deformation.

C Alternative definitions of the texture tensor

The texture tensor was introduced in the form of Eq. 10 and is modified as Eq. 13 in the main text. We also considered other possible forms of the texture tensor:

$$\hat{\mathrm{M}}^{(2)} = \frac{1}{N_{\mathrm{c}}} \sum_{i \in P} \frac{1}{2} \sum_{k}^{n_i} \omega_{ik} \boldsymbol{l}_{ik} \otimes \boldsymbol{l}_{ik} \;, \tag{S21}$$

$$\hat{\mathrm{M}}^{(3)} = \sum_{i \in P} \frac{1}{2n_i} \sum_{k}^{n_i} \omega_{ik} \boldsymbol{l}_{ik} \otimes \boldsymbol{l}_{ik} \;. \tag{S22}$$

$$\hat{\mathrm{M}}^{(4)} = \frac{1}{N_{\mathrm{c}}} \sum_{i \in P} \left(\frac{1}{2n_i} \sum_{k}^{n_i} \omega_{ik} \boldsymbol{l}_{ik} \otimes \boldsymbol{l}_{ik} \right) \;. \tag{S23}$$

$\hat{\mathbf{M}}^{(2)}$ is normalized by the number of cells in the ROI. $\hat{\mathbf{M}}^{(3)}$ and $\hat{\mathbf{M}}^{(4)}$ take into account the polygonal class of cells using a weighting factor proportional to $1/n_i$; $\hat{\mathbf{M}}^{(3)}$ is normalized by neighboring cells n_i to equalize the contribution of each polygonal cell. $\hat{\mathbf{M}}^{(4)}$ is further normalized by the cell number in the ROI, N_{c}, and is interpreted as a mean of individual cellular shape tensor $\frac{1}{2n_i}\sum_k^{n_i} \omega_{ik}\boldsymbol{l}_{ik} \otimes \boldsymbol{l}_{ik}$ over the ROI. All the proposed definitions of texture tensors possess a physical dimension of squared length, differing primarily in the normalization procedure based on the number of cells and their adjacent counterparts.

References

[1] B. Guirao, S. U. Rigaud, F. Bosveld, A. Bailles, J. Lopez-Gay, S. Ishihara, et al., Unified quantitative characterization of epithelial tissue development. Elife **4**:e08519 (2015). doi:10.7554/eLife.08519.001

[2] V. A. Lubarda. Constitutive theories based on the multiplicative decomposition of deformation gradient: Thermoelasticity, elastoplasticity, and biomechanics. Appl. Mech. Rev. **57**:95-108 (2004). doi:10.1115/1.1591000

[3] J. A. Blume, Compatibility conditions for a left Cauchy-Green strain field. J. Elast. **21(3)**:271-308 (1989). doi:10.1007/BF00045780

[4] A. Kaye, R. Stepto, W. Work, J. Aleman, A. Y. Malkin, Definition of terms relating to the non-ultimate mechanical properties of polymers (recommendations1998). Pure Appl. Chem. **70(3)**:701-754 (1998). doi:10.1351/pac199870030701

[5] S. Ishihara, P. Marcq, K. Sugimura, From cells to tissue: A continuum model of epithelial mechanics. Phys. Rev. E **96(2)**:022418 (2017). doi:10.1103/PhysRevE.96.022418

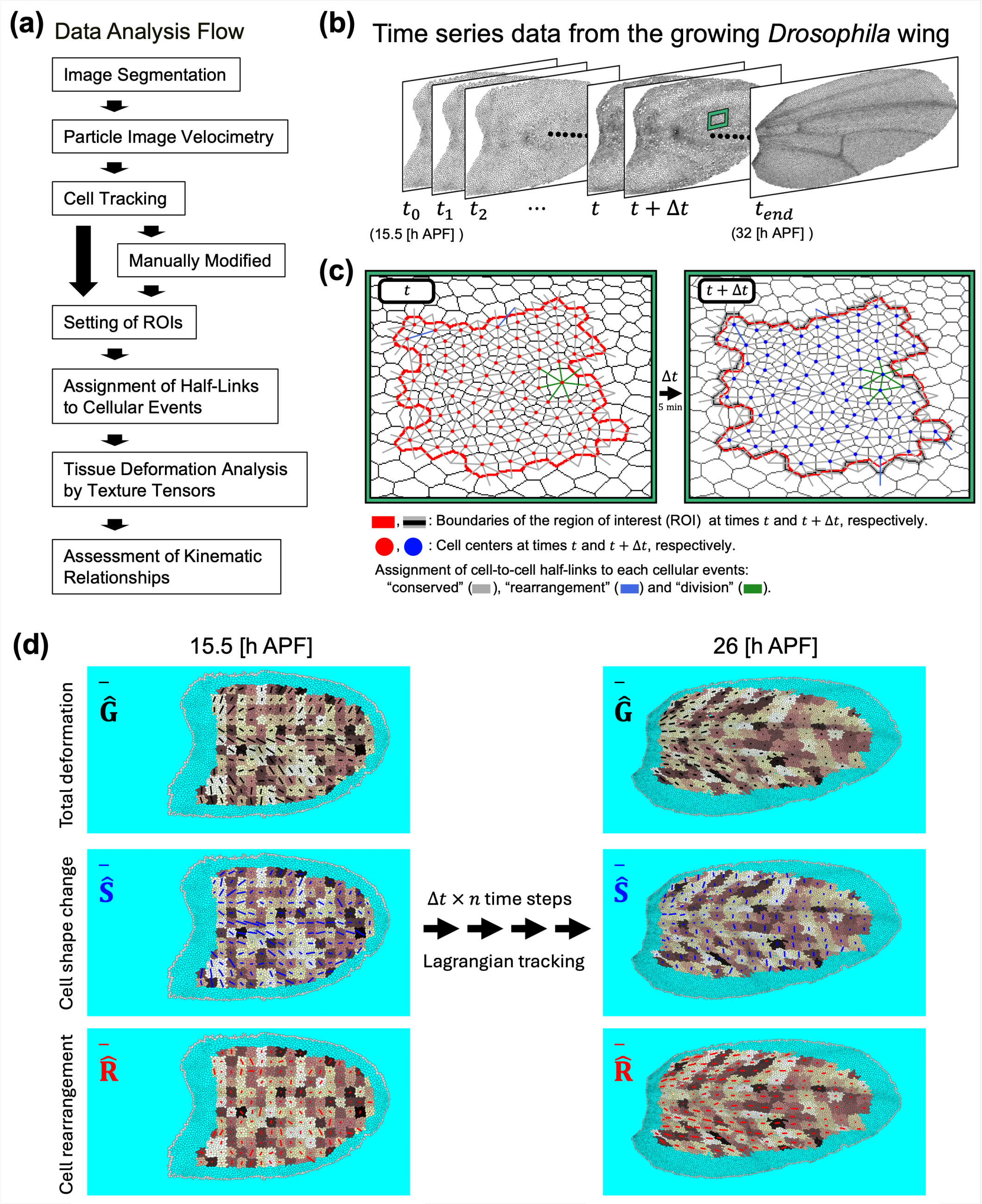

Figure S1. **Schematic diagram of data analysis.** (a) Flowchart of the data analysis procedure. (b) Skeletonized time-series image of growing *Drosophila* wings. (c) Changes in the texture in the region of interest (ROI) indicated by the closed red lines. Half-links between cell centers at times t and $t + \Delta t$ (red and blue filled circles, respectively) are shown. Color of half-links indicate their assignment to morphogenetic cell events (gray: conserved, blue: rearrangement, and green: division). The gray line represents the ROI boundary at each time point, while the red line at time $t+\Delta t$ is used for comparison with the ROI bounrady at t. (d) Spatial maps of mean-field quantities representing total deformation ($\hat{\mathbf{G}}$, black lines), cell shape change ($\hat{\mathbf{S}}$, blue lines), and cell rearrangement ($\hat{\mathbf{R}}$, red lines) across the entire wing at 15.5 and 26 h APF, with 15.5 h APF taken as the initial time. Bar shown in each ROI represents the deformation rates of the respective cellular events derived from the deviatoric component of each strain rate. The reference line in the top-left corner of each panel corresponds to a 1% change over 5 minutes. Time averaging was performed over 2-hour intervals.

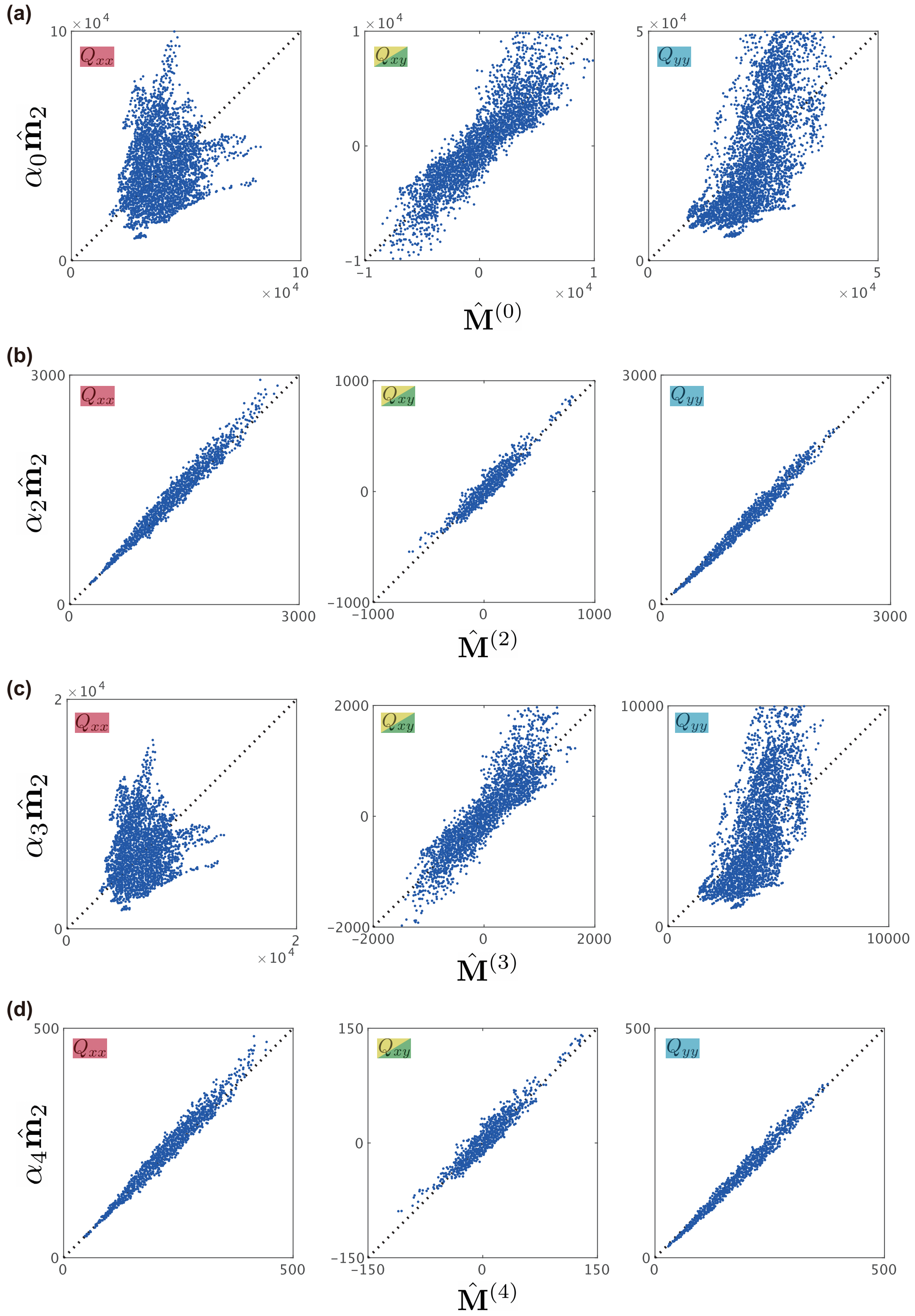

Figure S2. **Comparison of the second moments of cell shape for different definitions of cell shape tensors, $\hat{\mathbf{M}}^{(m)}$ ($m = 0, 2, 3, 4$).** (a–d) Each component of the tensors is shown in the respective panels. Individual dots represent data from single ROIs of 120 pixels × 120 pixels from the entire wing images (hereafter, the average is obtained over ROIs of this size unless noted otherwise). A scaling factor α was introduced as a fitting parameter for each measurement: (a) $\hat{\mathbf{M}}^{(0)}$: $\alpha = 196.61$, (b) $\hat{\mathbf{M}}^{(2)}$: $\alpha = 5.78$, (c) $\hat{\mathbf{M}}^{(3)}$: $\alpha = 32.39$, and (d) $\hat{\mathbf{M}}^{(4)}$: $\alpha = 0.95$.

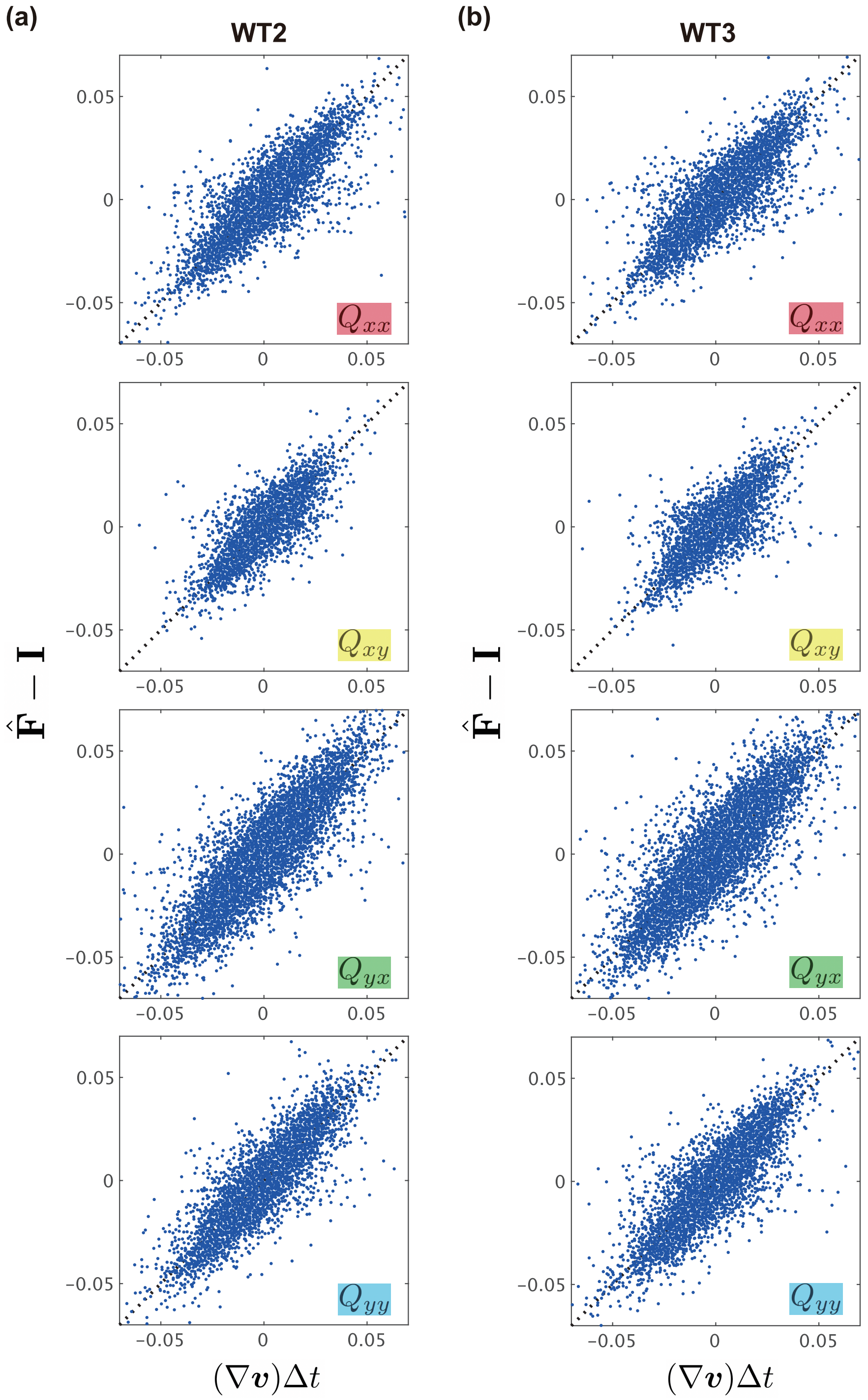

Figure S3. **Validation of strain rate tensors using data from additional samples.** (a, b) Data from WT2 (a) and WT3 (b) are analyzed and plotted similarly as in Fig. 3a.

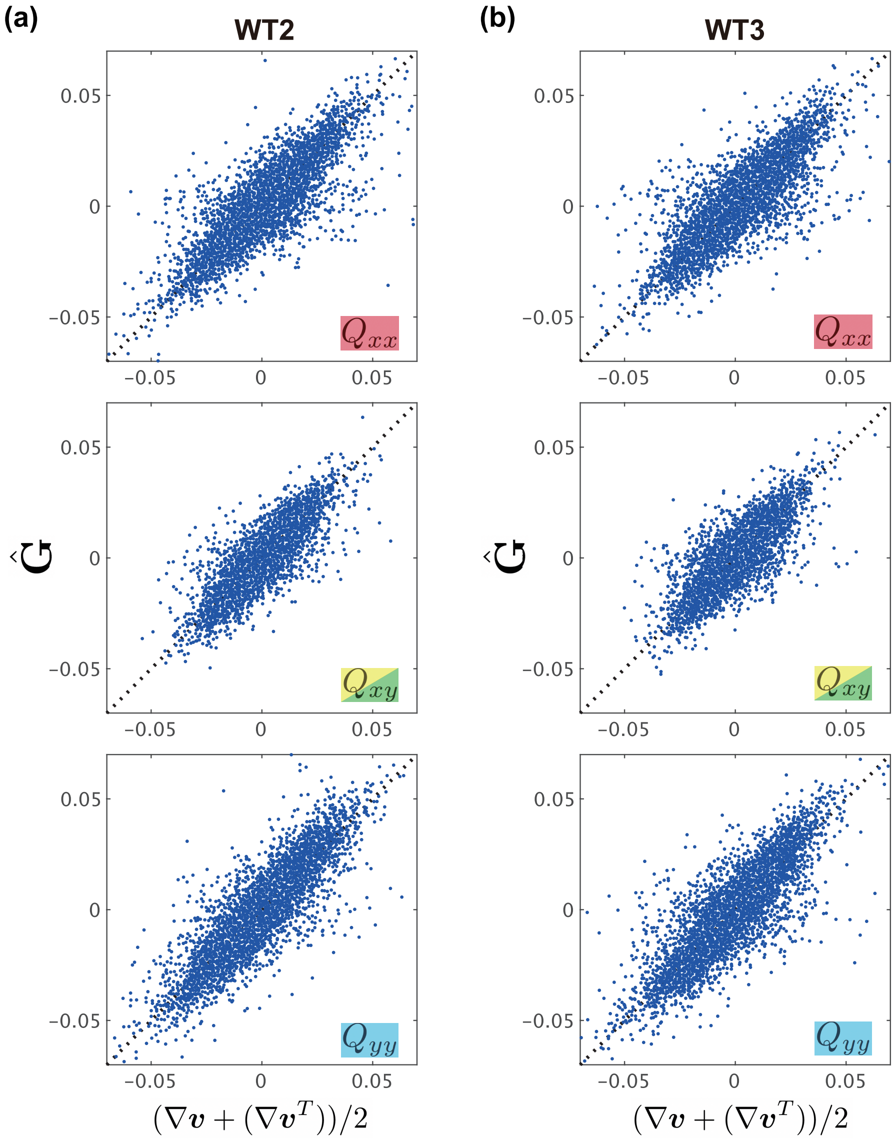

Figure S4. **Validation of the symmetric part of strain rate tensors using data from additional samples.** (a, b) Data from WT2 (a) and WT3 (b) are analyzed and plotted similarly as in Fig. 3b.

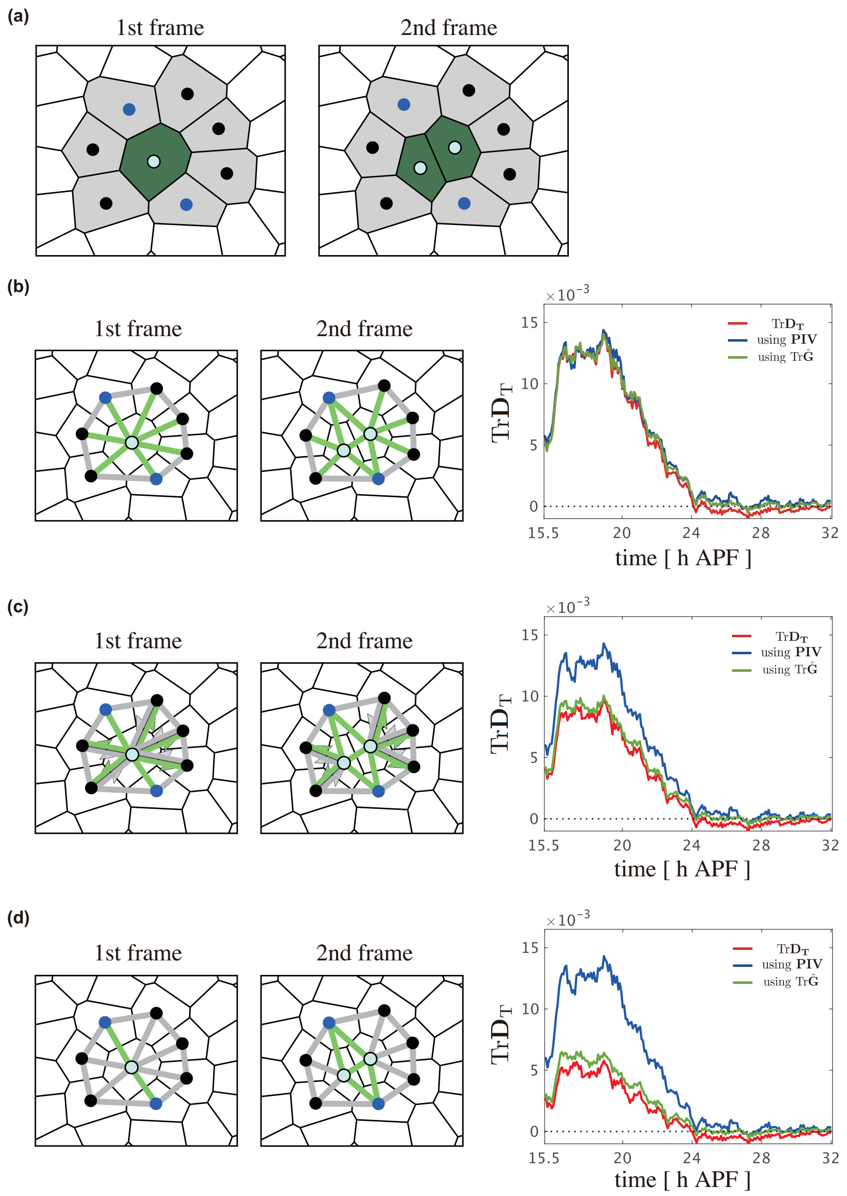

Figure S5. **Evaluation of assignment rules of half-links to each cellular event.** (a) Illustration of cell geometry change resulting from a cell division between the first to second timeframes. The dividing cells and their first-neighbor, non-dividing cells are distinguished by their colors (green and gray, respectively). The centers of the dividing cells are indicated by light blue points. In the case of first-neighbor, non-dividing cells, those with an increase in the number of adjacent cells are indicated with blue points, whereas those without such changes are represented by black points. (b-d) Tests of the cell number density equation (Eq. 8 in the main text) using different assignment rules. The left panels illustrate the assignment rules for half-links, with gray and green lines indicating half-links assigned to "conserved" and "division,". (b) Rule employed in the main text (the right panel is identical to Fig. 4b in the main text). (c, d) Alternative assignment rules for half-links involved in division (Sect. B). In (b) and (d), undirected links are assigned to the same cellular event for both directed half-links. In (c), half-links indicated by bi-directed arrows are assigned to different cellular events depending on their direction. The right panels indicate time-series data obtained by utilizing the corresponding assignment rules. The same whole-wing data (WT1) as in Fig. 4 was utilized, with data plotted similarly as in Fig. 4b. The values obtained by dividing the left-hand side of Eq. 8 by $\hat{\rho}$ are represented by blue and green lines and are compared with $\mathrm{Tr}\mathbf{D}_\mathrm{T}$, represented by the red line.

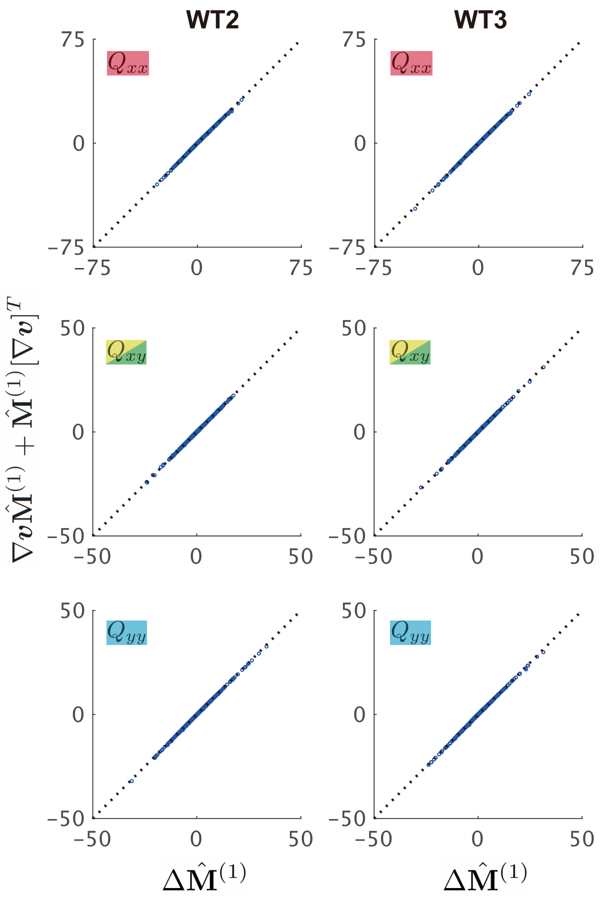

Figure S6. **Additional data for the validation of the kinematic equation $\hat{\mathbf{M}}^{(1)}$ in ROIs without topological deformation.** Data from WT2 and WT3 wings (left and right columns) are analyzed and plotted similarly as in Fig. 5a.

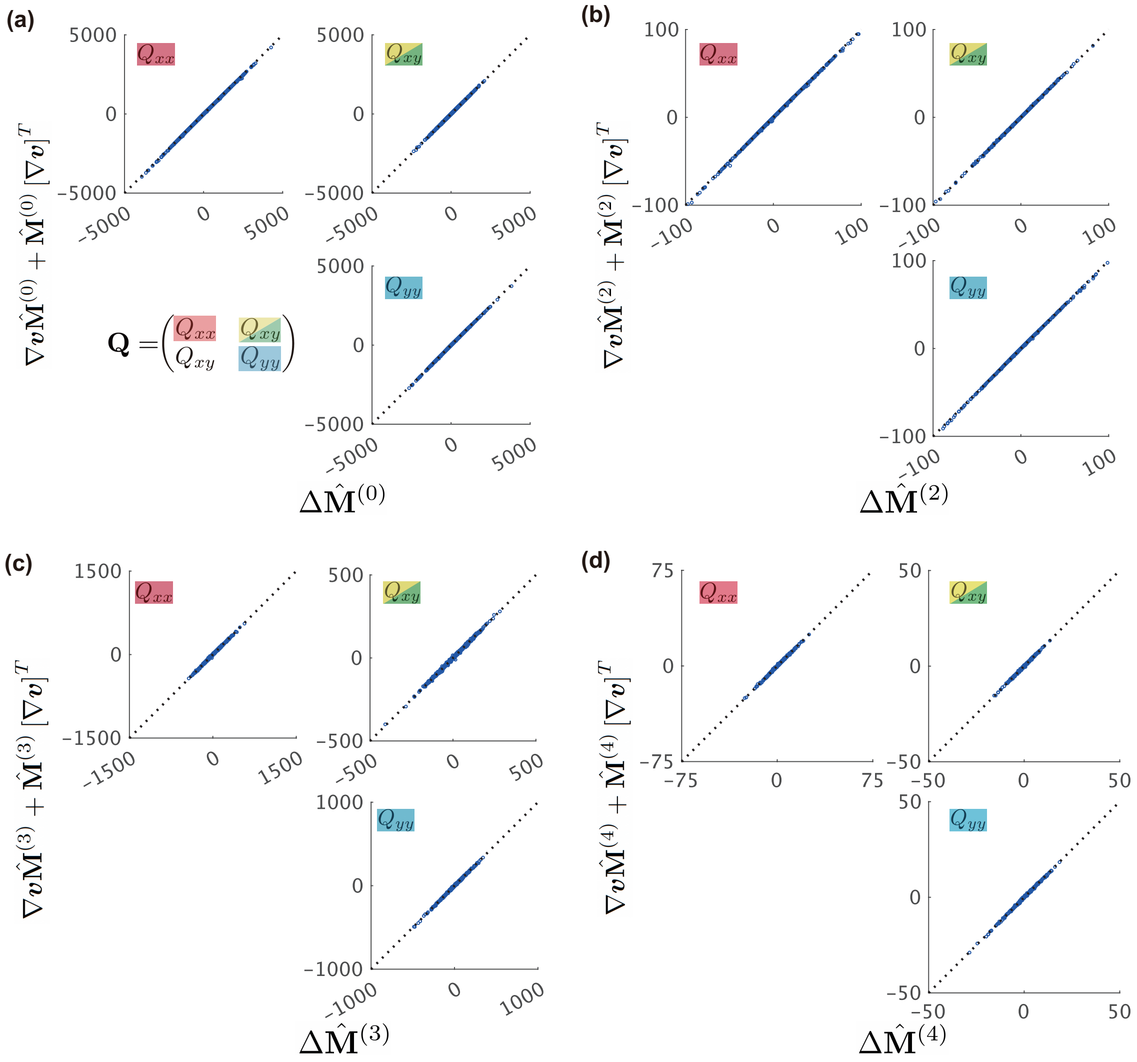

Figure S7. **Validation of kinematics for various definitions of $\hat{\mathbf{M}}$ in ROIs without topological deformation.** (a–d) The components of each tensor were evaluated for the following definitions of the texture tensor: (a) $\hat{\mathbf{M}}^{(0)}$, (b) $\hat{\mathbf{M}}^{(2)}$, (c) $\hat{\mathbf{M}}^{(3)}$, and (d) $\hat{\mathbf{M}}^{(4)}$. The same whole-wing data (WT1) as in Fig. 5a was utilized, with data plotted similarly as in Fig. 5a.

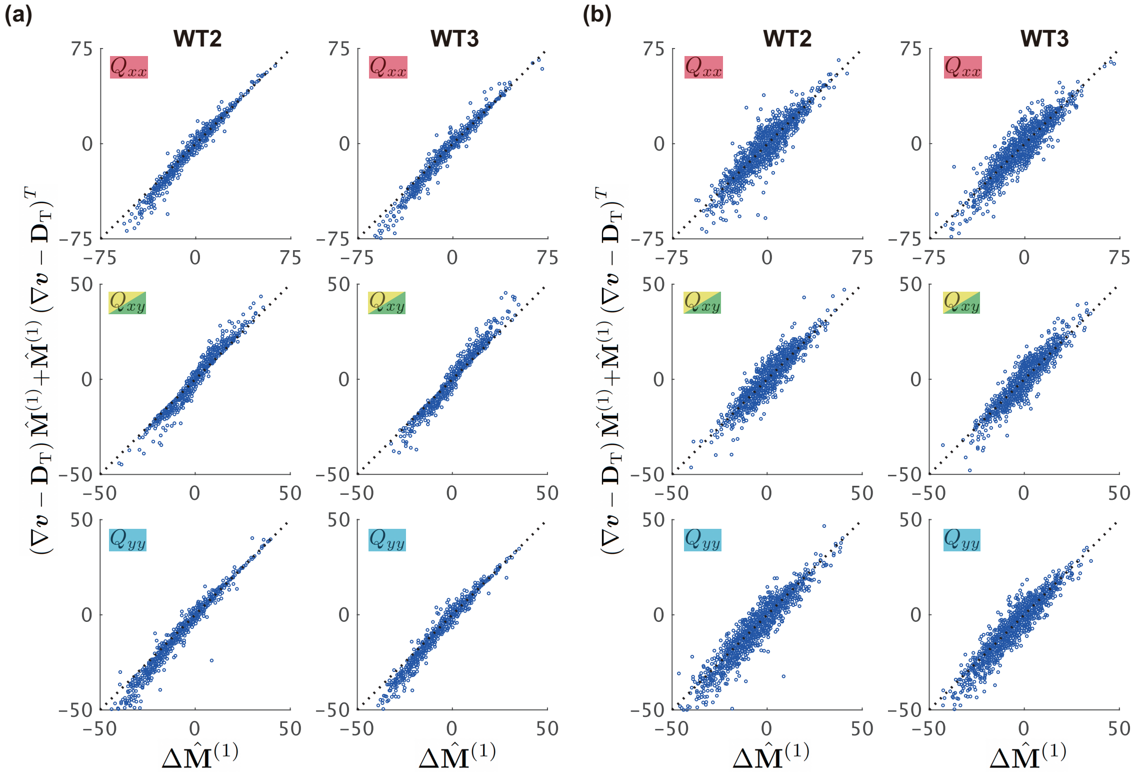

Figure S8. **Additional data for validation of the kinematic equation Eq. 7 using $\hat{\mathbf{M}}^{(1)}$.** (a, b) Data from WT2 and WT3 wings (left and right columns) were utilized to calculate $\hat{\mathbf{M}}^{(1)}$ using strain rate tensors based on $\hat{\mathbf{F}}$ (a) and PIV-measured $\nabla \boldsymbol{v}$ (b), respectively, as shown in Fig. 5b, c.

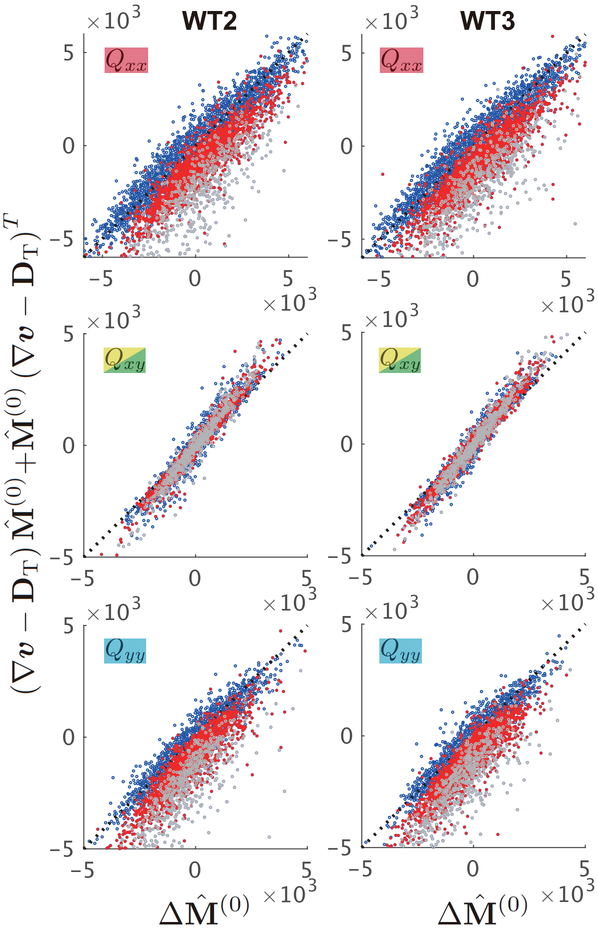

Figure S9. **Additional data for the validation of the kinematic equation utilizing $\hat{\mathbf{M}}^{(0)}$.** Data from WT2 and WT3 wings (left and right columns) were evaluated for the definitions in $\hat{\mathbf{M}}^{(0)}$ similarly to that in Fig. 5d. These point colors indicate the frequency of cell division—once (red) or multiple times (gray), or none (blue) within the ROI.

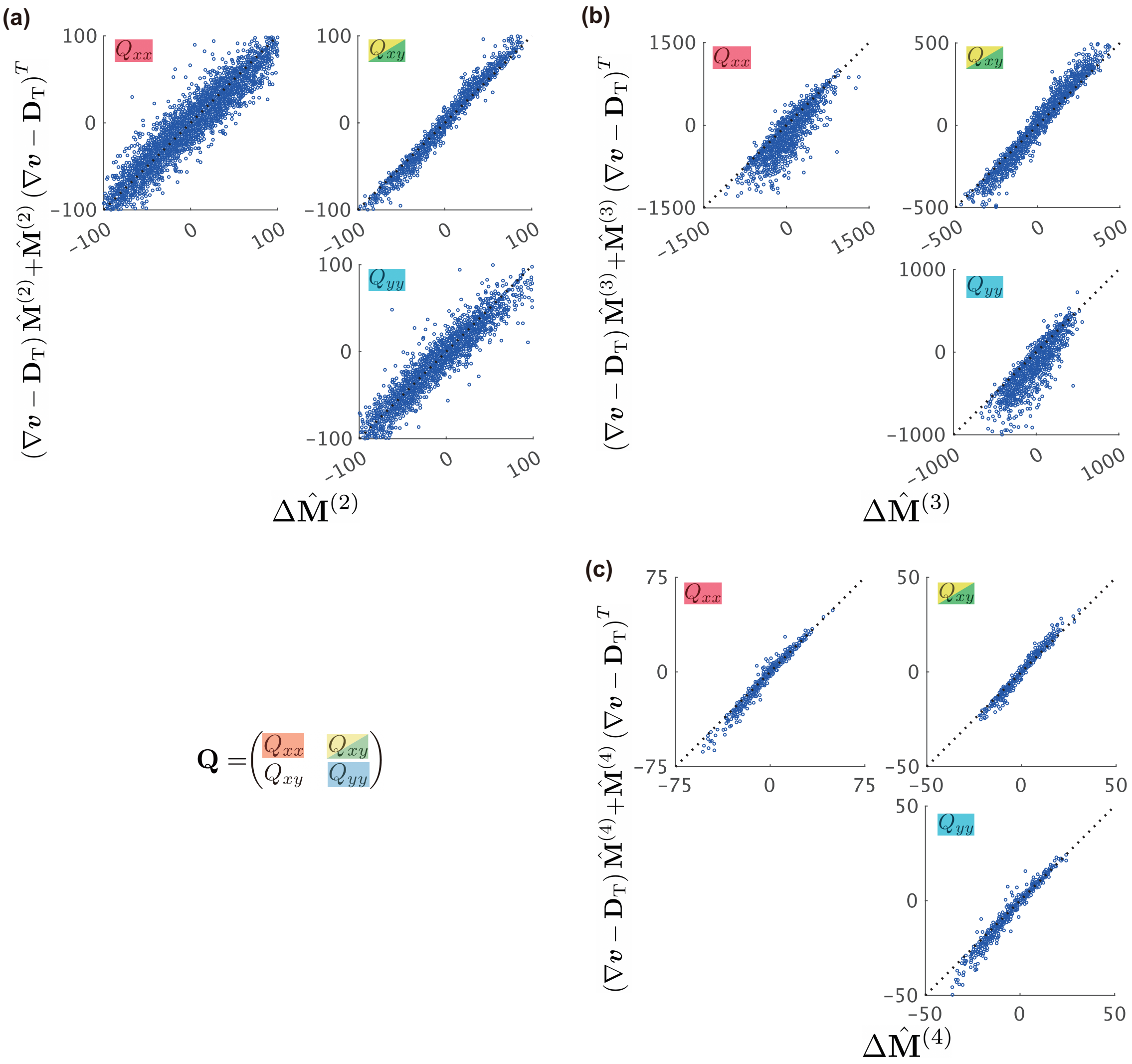

Figure S10. **Test of the kinematic equation for different definitions of $\hat{\mathbf{M}}$.** (a–c) The components of each tensor are evaluated for the following definitions of the texture tensor: (a) $\hat{\mathbf{M}}^{(2)}$, (b) $\hat{\mathbf{M}}^{(3)}$, and (c) $\hat{\mathbf{M}}^{(4)}$. The same whole-wing data (WT1) was utilized, as in Fig. 5b, with data plotted similarly as in Fig. 5b.

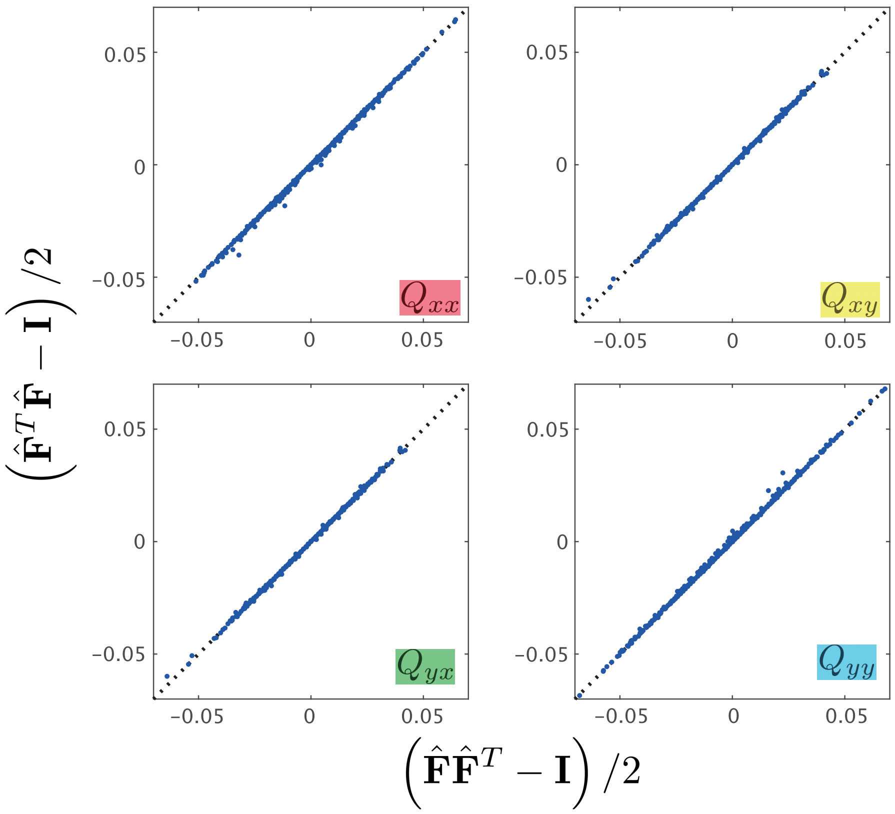

Figure S11. **Comparison of the different formulations of the Green-Lagrange strain tensor $\hat{\mathbf{E}}$.** The Green-Lagrange strain tensor utilized in this study (vertical axis) was plotted against that utilized in ref. [1] (horizontal axis).
